# High-Throughput Profiling of *Caenorhabditis elegans* Starvation-Responsive microRNAs

**DOI:** 10.1371/journal.pone.0142262

**Published:** 2015-11-10

**Authors:** Laura Garcia-Segura, Cei Abreu-Goodger, Armando Hernandez-Mendoza, Tzvetanka D. Dimitrova Dinkova, Luis Padilla-Noriega, Martha Elva Perez-Andrade, Juan Miranda-Rios

**Affiliations:** 1 Programa de Doctorado en Ciencias Biomédicas, Universidad Nacional Autónoma de México (UNAM), México, D.F., México; 2 Unidad de Genética de la Nutrición, Depto. de Biología Molecular y Biotecnología, Instituto de Investigaciones Biomédicas, UNAM e Instituto Nacional de Pediatría, México, D.F., México; 3 Unidad de Genómica Avanzada (Langebio), CINVESTAV, Irapuato, Guanajuato, México; 4 Centro de Investigación en Dinámica Celular, Universidad Autónoma del Edo. de Morelos, Cuernavaca, Morelos, México; 5 Departamento de Bioquímica, Facultad de Química, Universidad Nacional Autónoma de México, México, D.F., México; 6 Departamento de Virología, Facultad de Medicina, Universidad Nacional Autónoma de México, México, D.F., México; National Institutes of Health, UNITED STATES

## Abstract

MicroRNAs (miRNAs) are non-coding RNAs of ~22 nucleotides in length that regulate gene expression by interfering with the stability and translation of mRNAs. Their expression is regulated during development, under a wide variety of stress conditions and in several pathological processes. In nature, animals often face feast or famine conditions. We observed that subjecting early L4 larvae from *Caenorhabditis elegans* to a 12-hr starvation period produced worms that are thinner and shorter than well-fed animals, with a decreased lipid accumulation, diminished progeny, reduced gonad size, and an increased lifespan. Our objective was to identify which of the 302 known miRNAs of *C*. *elegans* changed their expression under starvation conditions as compared to well-fed worms by means of deep sequencing in early L4 larvae. Our results indicate that 13 miRNAs (miR-34-3p, the family of miR-35-3p to miR-41-3p, miR-39-5p, miR-41-5p, miR-240-5p, miR-246-3p and miR-4813-5p) were upregulated, while 2 miRNAs (let-7-3p and miR-85-5p) were downregulated in 12-hr starved vs. well-fed early L4 larvae. Some of the predicted targets of the miRNAs that changed their expression in starvation conditions are involved in metabolic or developmental process. In particular, miRNAs of the miR-35 family were upregulated 6–20 fold upon starvation. Additionally, we showed that the expression of *gld-1*, important in oogenesis, a validated target of miR-35-3p, was downregulated when the expression of miR-35-3p was upregulated. The expression of another reported target, the cell cycle regulator *lin-23*, was unchanged during starvation. This study represents a starting point for a more comprehensive understanding of the role of miRNAs during starvation in *C*. *elegans*.

## Introduction

When cells are deprived of nutrients, they respond to starvation by changes in intracellular signaling, in order to enhance their chances of survival [1]. One such response is metabolism modulation by activating catabolic pathways and suppressing anabolic ones, generating necessary metabolites to maintain core cellular activities [2]. If homeostasis cannot be re-established, a new gene expression program is enforced to try to escape cell death. MicroRNAs (miRNAs) are thought to help maintain homeostasis and/or reprogram gene expression [3].

miRNAs are non-coding, short RNAs of approximately ~22 bases that modulate stability and translational capacity of their mRNA targets [4]. More than half of all mammalian mRNAs are predicted targets of miRNAs [5]. Considering that the number of human miRNAs already reported is 2,603 (miRBase Release 20 [6]), and maybe reaching over six thousand, according to a recent analysis of sRNA-seq datasets from 13 human tissue types [7], they outnumber cellular kinases and phosphatases (more than 500 kinases and 150 phosphatases have been predicted in the human proteome), emphasizing their importance in regulation. MiRNAs also play key roles in mediating stress responses [8]. Paradoxically, inactivation of most individual miRNAs in flies and worms has no effect on viability or development when assayed under standard laboratory conditions [9–11]. In contrast, they seem to be indispensable when mutant animals are subjected to stress conditions: *e*.*g*. miR-7 knockout flies are unable to properly develop their eyes if the flies are housed in an environment that shows cyclic temperature variations [12]. Stress conditions can produce dramatic changes in miRNA biogenesis, subcellular localization, activities of miRNA-protein complexes and the expression of their targets [3].

Several fundamental phenomena were discovered in *C*. *elegans* such as programmed cell death, RNAi and endogenous regulation by miRNAs. *C*. *elegans* is a relatively simple animal formed by 959 somatic cells in the hermaphrodite and is widely used as an experimental model due to the large amount of genetic tools and available mutants. It has a short generation time, is easy to culture and has a fully sequenced genome. Because it is completely transparent, it allows direct visualization of gene expression with reporters like GFP. In addition, it is simple to perform high-throughput studies on gene expression using knockouts or knockdowns by RNAi [13].

*C*. *elegans* develops from embryo to adult in 3 days, going through 4 different larval stages (L1, L2, L3 and L4). The adults can live for up to 3 weeks under favorable conditions [14]. Like many other animals, *C*. *elegans* responds to starvation by entering developmental arrest at different stages of its life cycle. When embryos hatch in the absence of food, they enter an L1 diapause and can survive for about two weeks [15]. If late L1 worms are subjected to starvation, they enter an alternative stage called dauer larvae (or dauer diapause), in which development is arrested between L2 and L3 larval stages [16]. In the dauer state, larvae do not feed or move, show a low respiratory rate, lower their ATP consumption and are highly resistant to external conditions [16]. When food is available, worms exit diapause, resume larval development into the adult stage with full reproductive capacity and a normal life span.

The participation of miRNAs in development has been extensively studied, but information of how they are involved in other cellular processes is still scarce. Recently, a combination of genetics, biochemistry and bioinformatics has been used to elucidate the physiological function and biological relevance of several miRNA-target interactions, as well as to derive miRNA-target interaction networks that show how miRNAs are involved in robust cellular responses to different environmental conditions [17]. Different diets, caused by augmented intake or deficiency of nutrients such as carbohydrates, vitamins, fatty acids, and amino acids are also known to produce changes in miRNA expression [18]. Several miRNAs have been reported to change their expression under starvation conditions. For example, miR-71 was shown to be involved in L1 starvation survival as it interacts with several target genes from the Insulin/IGF-1 signaling (IIS) pathway, in addition to its role in longevity [19–22]. Under dietary restriction conditions, multiple miRNAs changed their expression level, including miR-71 and miR-228, among others [23,24].

Fasting response studies have mostly focused on proteins, such as changes in activity and/or abundance of metabolic enzymes, signaling pathways or transcriptional factors. These studies have been performed only in a limited number of isolated tissues (muscle, liver, etc), and not on the whole organism, that allows for a more convenient analysis of systemic effects. Nutrient availability has profound effects on gene expression that involve the participation of different pathways. Such a complex activity needs a set of regulatory factors that coordinate the responses to starvation at the organismal level. miRNA-mediated regulation, in conjunction with other mechanisms of gene regulation, such as transcriptional regulation and protein degradation, participate to enhance the robustness of the response to different physiological conditions [25]. In particular, miRNAs have the ability to adjust the expression of a large number of proteins as each miRNA is able to target many different mRNAs, and each mRNA could be targeted by several miRNAs at once [26]. Additionally, the expression of the miRNAs is tightly regulated in response to different stress conditions [27,28]. Furthermore, the response mediated by miRNAs occur in the timescale of hours, producing changes in metabolism and gene expression that allow the organisms to circumvent different challenges [29–31].

Given the above-mentioned characteristics associated to miRNA-mediated regulation, we think they could be key participants in the response to nutrient-deficiency. Recently, Larance *et al*. [32] reported that approximately 5,000 proteins changed their abundance under starvation conditions in *C*. *elegans*, most of them involved in central metabolic pathways, and others including chromatin-associated proteins. It is possible that some of the changes observed in the proteome are the result of regulation performed by miRNAs. Here, we addressed the question of how miRNAs participate in the response to food deprivation in *C*. *elegans* L4 larvae. We found that several miRNAs changed their expression under starvation conditions, among them the miR-35-41 cluster that was unregulated. MiRNAs of this cluster are involved in regulating the expression of *gld-1* and *lin-23*, whose products are important for gonad formation and ovogenesis, and for cell cycle control, respectively.

## Materials and Methods

### Strains and Culture

The wild type strain N2 of *C*. *elegans* was grown under standard conditions at 18^°^C and fed with *Escherichia coli* OP50 and cholesterol [33]. The worms were synchronized by killing all the larval and adult worms by immersion in sodium hypochlorite, a condition in which only eggs can survive [33]. After synchronization, worms were seeded on a Petri dish and fed with *E*. *coli* OP50 until they reached early L4 larval stage; half of the larvae were washed with M9 buffer to eliminate all bacteria, after the washes the larvae were incubated on a Petri dish without bacteria, while the other half were normally fed. After 12 hrs, both samples (well-fed and 12-hr starved) were washed with M9 buffer (42.26 mM Na_2_HPO_4_; 22.04 mM KH_2_PO_4_; 85.56 mM NaCl; 0.87 mM MgSO4).

### Length measurement

One drop of M9 buffer-containing well-fed and 12-hr starved early L4 larvae were laid on a Neubauer chamber for length measurements; 30 larvae were measured for each condition, and photographed under a Nikon SMZ800 stereoscopic microscope.

### Body fat assessment

Well-fed and 12-hr starved early L4 larvae were washed three times in M9 buffer. The pellets of larvae were resuspended in 120 ul of PBS 1X and an equal volume of buffer MRWB 2X (160 mM KCl; 40 mM NaCl; 14 mM Na_2_EGTA; 0.2% β-mercaptoethanol), containing 2% of paraformaldehyde. The larvae were agitated at 1000 rpm for 1 hr at room temperature, washed 3 times with PBS 1X to eliminate the paraformaldehyde. Samples were suspended in isopropanol 60% to dehydrate, incubated for 15 min at room temperature and were suspended in 60% Oil-Red-O stain (prepared as follows: from 0.5 g/100 mL isopropanol stock solution equilibrated for several days, freshly diluted with 40% water, allowed to sit 10 min and filtered through 0.2 to 0.4 μm). Larvae were photographed under a 20X objective in an Olympus microscope BX51W1 coupled to a Disk Spinning Unit.

### Brood size quantification

Well-fed L4 larvae or larvae subjected to starvation as described previously, were placed on individual 3-cm NGM plates and fed with *E*. *coli* OP50 strain (n = 21, three replicates for each condition). After three days, larvae produced by each worm were counted under a stereoscopic microscope (Nikon SMZ800).

### Gonad size estimation

Well-fed and 12-hr starved L4 larvae were washed three times in M9 buffer; after the last wash, the supernatant was drained and 300 ul of -20°C methanol was added and left for 5 min. Then 200 ul of PBS-T (PBS 1X, 0.1% Tween 20) were added and centrifuged at 3000 rpm for 1 min. Two washes with 500 ul of PBS-T were performed. Finally, one drop containing the worms plus one drop of 100% glycerol were placed on a microscope slide and covered with a cover slip. The worms (n = 5) were observed under an Olympus Laser Scanning Confocal Microscope (100X objective). Area estimation was calculated with the ImageJ program (http://imagej.nih.gov/ij/), and reported as pixels/micron^2^.

### Lifespan assessment

Well-fed and 12-hr starved early L4 larvae were fed until they reached the adult stage, then 100 worms from each condition were seeded on a Petri dish. Dead worms were counted daily, and withdrawn with a platinum wire. To get rid of embryos and L1 larvae, the Petri dishes were washed every other day.

### RNA isolation, cDNA library preparation and Illumina deep sequencing

Small RNAs were purified and size-selected by using the miRNeasy mini kit and minelute columns following manufacturer’s instructions (Qiagen). The cDNA libraries were prepared from 5 ug of RNAs <200 nt. Preparation of cDNA libraries for the Illumina deep sequencing experiment was performed using the DGE-Small RNA Sample Prep Kit ver. 1.0 (Illumina) according to the manufacturer’s instructions. Briefly, RNAs between 20 and 30 nt in size were purified and ligated to adapters and amplified by RT-PCR. Purified DNA was captured on an Illumina flow cell for cluster generation. Libraries were sequenced for 36 cycles on an Illumina Genome Analyzer IIx using Illumina’s protocols for single-end reads (DGE-Small RNA Cluster Generation Kit and 36 Cycle Solexa Sequencing Kit). The raw deep-sequencing data and processed data are available from GEO GSE67711.

### *C*. *elegans* genome and non-coding RNA annotation

The full genome, the protein-coding and non-coding transcripts annotation were downloaded from WormBase version WS235 in FASTA format [34]. Sequences for all known *C*. *elegans* miRNA hairpins were obtained from miRBase version 20 [6]. Mature sequences were also obtained from this database, but a Perl script was used to extend them by 3 bases at the 5’ end and 5 bases at the 3’ end, when permitted by the length of the hairpin. This extension facilitates sRNA-seq reads to map directly to mature miRNAs. All FASTA sequences were then concatenated and converted into an index for bowtie version 0.12.9 [35].

### Processing and mapping sRNA-Seq reads

An artificial hexamer (ACATCG) was present at the 5’ end of 6–8% of all reads, so a custom Perl script was used to trim these occurrences. We then used Reaper to process the FASTQ files [36], and remove the Illumina sRNA 3’ adapter sequence (ATCTCGTATGCCGTCTTCTGCTTGC). Since we were interested in miRNAs, only cleaned reads between 16 and 28 nucleotides were kept. These sequences were collapsed to unique reads using tally [36], assigning their total counts to each sequence identifier. The resulting FASTA files were mapped to the concatenated sequences described in the previous section using bowtie [35], searching for end-to-end hits with at most 2 mismatches, and allowing reads to hit up to 100 different locations. All further processing was performed with *ad hoc* shell and R scripts using several packages from the Bioconductor project [37]. In particular, reads were first assigned to a class with the following preference: miRBase mature microRNA > miRBase microRNA hairpin > WormBase non-coding transcript > WormBase coding transcript > WormBase intergenic. Reads assigned to a microRNA hairpin are those that mapped outside the annotated mature regions; we expect these to mostly represent reads coming from the loop region, a byproduct of miRNA biogenesis. Once a read was assigned to a class, the observed counts for each unique read were divided between any multiple locations within the same class. Any read that ended with a divided count of less than 1 was ignored. The collapsed sequences were also processed with the miRanalyzer free-web server tool [38] to predict the presence of previously unknown miRNAs candidates, using the default parameters, allowing 1 mismatches, and a threshold of the posterior probability to consider a new miRNA of 0.9.

### Differential expression of miRNAs

For differential expression analysis we only considered reads that preferentially mapped to miRBase sequences. We only considered miRNA regions with at least 1 reads per-million from one of the libraries. These were tested for differential expression using the edgeR package [39], setting the common dispersion to 0.1 (since we did not have biological replicates) and normalizing with the TMM method. False Discovery Rates were calculated using the Benjamini & Hochberg procedure [40].

### Predicted miRNA-mRNA target interactions

We searched for predicted miRNA targets involved in metabolism or development, using microRNA.org [41], miRanda [42] and TargetScan [43] websites. These results are presented in S2 and S3 Tables, with references indicating the involvement of the target genes in metabolism or development included in S1 File.

### qRT-PCR

Stem-loop qRT-PCR was used for the quantification of miRNAs, to provide an enhanced sensitivity and specificity as compared to linear primers [44,45]. Stem-loop RT primers include a modification to provide the Universal ProbeLibrary Probe #21 sequence binding site into the primer stem region [46]. All reactions were carried out in triplicate, both biological and technical. Primers used for Reverse Transcription of miR-35-3p, miR-36-3p, miR-39-3p, miR-240-5p, miR-246-3p, and miR-58-3p were RTCEL35, RTCEL36, RTCEL39, RTCEL240, RTCEL246, and RTCEL58, respectively (S1 Table). Primers used for Reverse Transcription of *lin-23*, *gld-1* and *β-actin* were LIN23-reverse, GLD-reverse and ACT-reverse, respectively (S1 Table). For Reverse Transcription of miRNAs (miR-35-3p, miR-36-3p, miR-39-3p, miR-240-5p, miR-246-3p, and miR-58-3p) and mRNAs (*gld-1*, *lin-23* and *β-actin*), a total of 2000 ng and 300 ng were used, respectively. The Reverse Transcription mix contained RNA, oligonucleotide (250 fmol) and water for a final volume of 4 ul, and was incubated at 70°C for 5 min. Then, dNTPs, buffer for RT and Reverse Transcriptase (RevertAid H Minus Reverse Transcriptase, Fermentas) were added and the mix was incubated at 37°C for 5 min, 42°C for 1 hr and 70°C for 10 min.

For miRNA´s qPCR quantification, a total of 2000 ng of cDNA was used for each reaction (each reaction was done in duplicate). Primers pairs were FCEL35/UPR, FCEL36/UPR, FCEL39/UPR, FCEL240/UPR, FCEL246/UPR, and FCEL58/UPR for miR-35-3p, miR-36-3p, miR-39-3p, miR-240-5p, miR-246-3p, and miR-58-3p, respectively (S1 Table). Primers and the hydrolysis probe (Universal ProbeLibrary Probe #21, Roche) were used at 1 μM and 0.1 μM, respectively. Reactions were done in a final volume of 18 ul, using Master Mix 1X (LightCycler TaqMan Master) in a LighCycler 2.0 (Roche) equipment. The program used for qPCR was: 1 cycle (at 95°C for 2 min), 40 cycles (at 94°C for 15 secs, 45°C for 30 secs, 70°C for 30 secs), 1 cycle (at 70°C for 15 min). The expression of miR-58-3p was used as an endogenous control. The quantification of miR-35-3p, miR-36-3p, miR-39-3p, miR-240-5p, and miR-246-3p expression relative to miR-58-3p was calculated as in Pfaffl, 2001[47].

For *gld-1*, *lin-23* and *β-actin* mRNA qPCR quantification, we used 100 ng of cDNA. Primer pairs were LIN23forward/LIN23-reverse, GLD-forward/GLD-reverse, ACT-forward/ACT-reverse, respectively (S1 Table). Primers were used at a concentration of 50 nM and SYBR Green PCR Master Mix (Applied Biosystems) was used. Reactions were run in a StepOne Applied Biosystems equipment. The program used for qPCR was: 1 cycle (at 95°C for 2 min), 40 cycles (at 95°C for 15 secs, 50°C for 30 secs, 70°C for 1 min), 1 cycle (at 70°C for 15 min). The *β-actin* gene was used as the endogenous control. The relative expression ratio of *gld-1* and *lin-23* mRNAs relative to *β-actin* mRNA expression was calculated as previously described[47].

### Statistics

In this study, statistical analyses were performed using GraphPad Prism software (San Diego, CA). A Wilcoxon Signed Rank Test was used to compare length differences between well-fed and 12-hr starved worms. For brood size analysis, a Student´s t-test was performed. The significance of analysis of longevity was performed using Kaplan-Meier survival curves method and Log-rank (Mantel-Cox) test. Differential (upregulated or downregulated) expression of miR-35-3p and target mRNAs *gld-1* and *lin-23* (qRT-PCR) was also analyzed using Wilcoxon Signed Rank Test. A *p*-value ≤ 0.05 was considered to be statistically significant.

### Data deposition

All the raw deep-sequencing data and processed data are available from the NCBI Gene Expression Omnibus (GEO) database under accession number GSE67711.

## Results and Discussion

### Fasting for 12-hrs has dramatic effects on the physical appearance, lipid accumulation, brood size, gonad size, and lifespan in early L4 larvae of *C*. *elegans*

**Fig 1:**
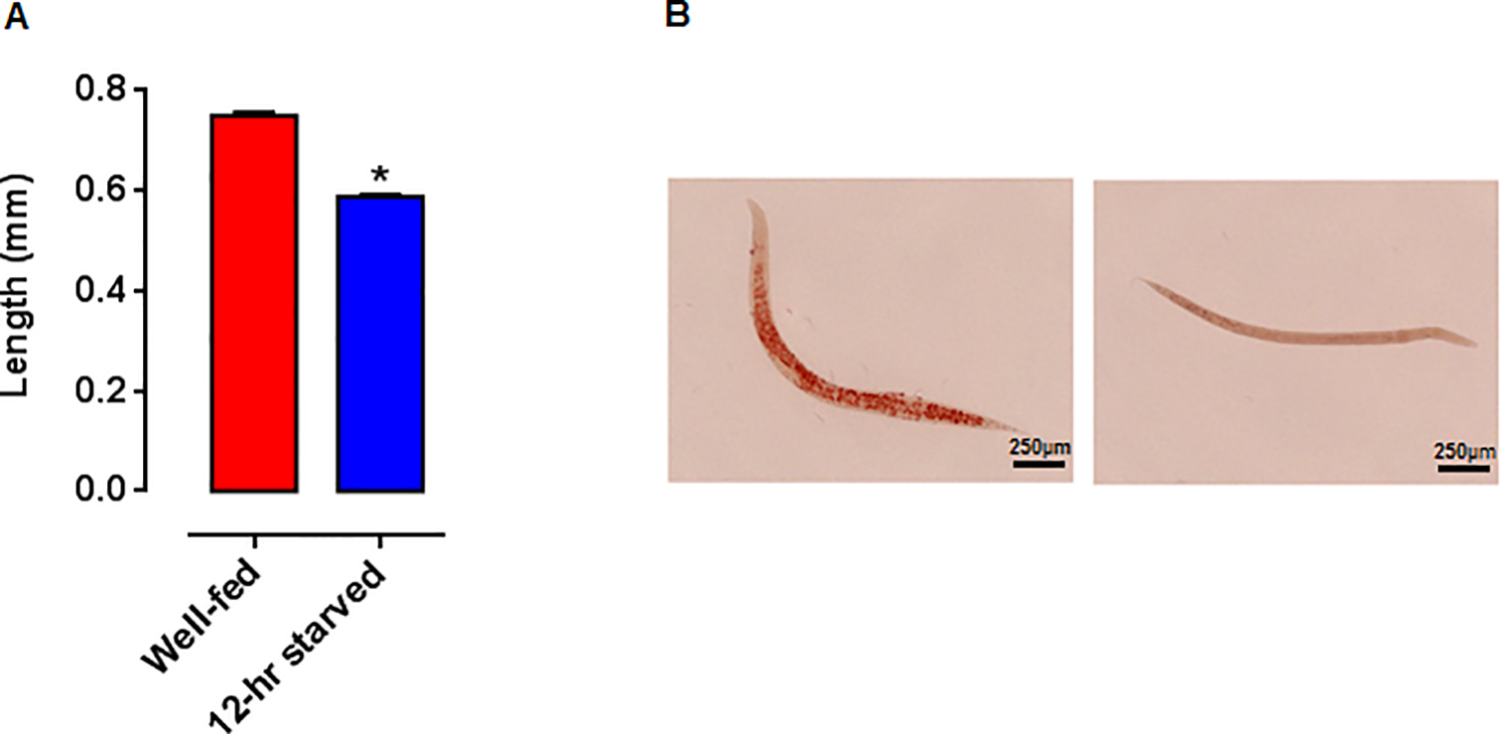
Physical aspect, length and lipid content of well-fed and 12-hr starved early L4 larvae. (A) Length of well-fed and 12-hr starved early L4 larvae, n = 30 early L4 larvae per group, mean ± standard error of the mean, p < 0.0001 according to a Wilcoxon Signed Rank Test. (B) Lipid content of well-fed and 12-hr starved early L4 larvae stained with Oil-Red-O (20X objective).

We determined the effect of fasting for 12-hrs on C. elegans larvae. Starved-larvae were thinner and shorter in length compared to well-fed animals. The length of the animals was 0.75 ± 0.01 mm and 0.59 ± 0.01 mm (mean ± standard error of the mean) for the well-fed and starved animals, respectively (Fig 1A).

**Fig 2:**
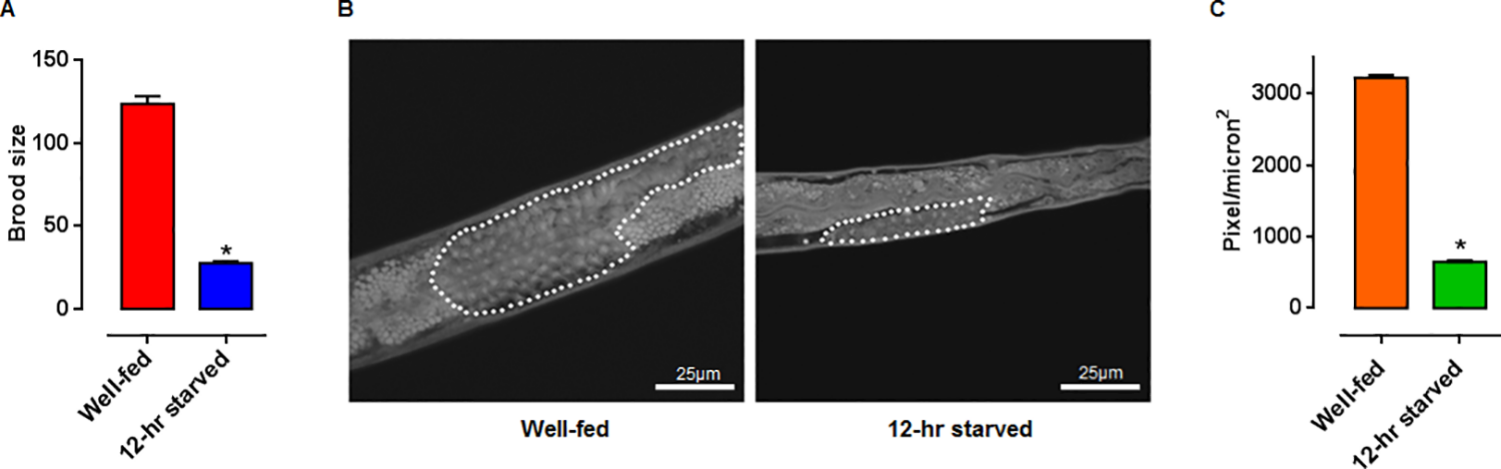
Effect of a 12-hr starvation on brood size and gonad formation. **(**A) Total larval progeny per hermaphrodite at day 3, n = 21, p-value<0.0001 according to a Student’s t-test. (B) Representative images of the proximal gonad from 12-hr starved vs. well-fed early L4 larvae, as seen by DIC microscopy (100X objective). (C) Estimated area of the proximal gonad, n = 5 early L4 larvae per group, mean ± SEM, *p*-value <0.0079 according to a Wilcoxon Signed Rank Test.

Because animals subjected to even 4–6 hrs of fasting can deplete their fat reserves [48], we analyzed the amount of lipids in well-fed vs. 12-hr starved animals. Fasted animals showed a significant decrease in the amount of lipids, maybe as a consequence of the catabolism of lipids as a mechanism to re-establish homeostasis (Fig 1B). A similar result has been reported for starved worms in feeding defective mutants [49]. These results show that starvation of early L4 larvae has dramatic effects on the physical appearance and the accumulated lipids in the worm. We also observed a very dramatic effect on the fertility of L4 larvae subjected to a 12-hr starvation, as the progeny diminished by 78% in contrast to the well-fed group. The brood size was 123 ± 5 and 27 ± 1 larvae, mean ± SEM) for the well-fed and 12-hr starved worms, respectively) (Fig 2A). In general, a reduction in reproductive ability has been documented as a consequence of the lack of food, since energy is allocated into cellular processes needed for survival [50]. In particular, fecundity has been found to correlate with the amount of bacterial food source in *C*. *elegans* [51]. Several pathways have been shown to be involved in such phenomena, such as the Insulin/IGF, mTOR (let-363), and cytochrome P450 (DAF-9/CYP450), steroid hormone Δ7-dafachronic acid (DA), and nuclear hormone receptor NHR-8 [52]. This result displays a large effect on fertility produced by starvation.

Food-deprivation of L4 larvae can result in L4 or adult arrest, adult matricide of bagging, adult reproductive diapause and eugenic germ line starvation response, causing defects in gametogenesis, gonad development, reproductive competence and longevity [53,54]. Which of the different outcomes arises depends on the precise time of starvation onset and its duration [53,54]. We found that after a 12-hr starvation treatment, the area occupied by the proximal gonad was dramatically decreased (Fig 2B and 2C). A diminished gonad could represent one of the many factors that could explain the reduction in progeny that we observed (Fig 2A). These results are consistent with previous reports, as when L4 larvae were subjected to starvation, a decrease in the size of their gonad was observed [54].

**Fig 3:**
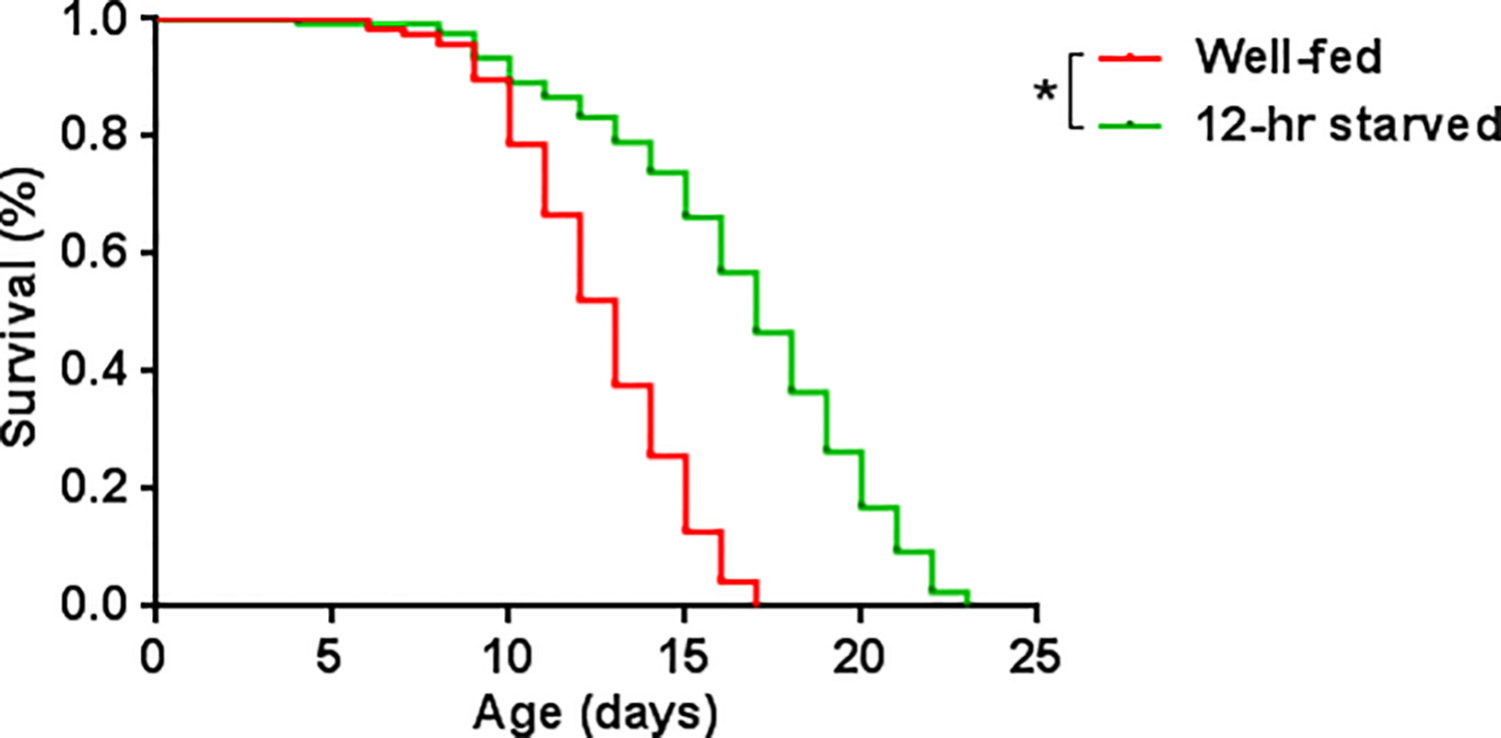
Kaplan-Meir survival curves of well-fed (n = 117) and 12-hr starved (n = 112) early L4 larvae. Starved worms showed a significantly increased lifespan (*) compared to well-fed early L4 larvae, according to a log-rank (Mantel-Cox) test (*p* < 0.0001).

Another aspect that we wanted to examine was if starvation for 12 hrs produced changes in the worm’s lifespan. Dietary restriction, a reduction in caloric uptake without malnutrition, can increase the lifespan in different organisms such as yeasts, worms, flies, rodents, non-human primates and possibly even in humans [55]. We found that early L4 larvae fasted for 12 hrs showed an increased lifespan (16.9 d), compared to that of well-fed worms (12.4 d). The increase was 36% (Fig 3). This amount is comparable in magnitude to the lifespan extension provoked from a complete removal of food during adulthood in *C*. *elegans* [56].

In summary, a 12-hr starvation period during early L4 larvae produced worms that were thinner and shorter than well-fed animals, with a decreased lipid accumulation, diminished progeny, reduced gonad size, and an increased lifespan.

### Deep sequencing analysis of miRNAs from early L4 *C*. *elegans* subjected to food starvation for 12 hrs.

**Fig 4:**
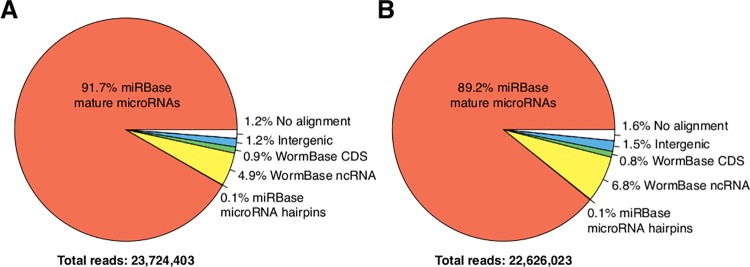
Functional classification of sRNA-seq reads. Reads were classified according to the type of sequence to which they mapped (see Materials and Methods). (A) Well-fed, and (B) 12-hr starved early L4 larvae.

**Fig 5:**
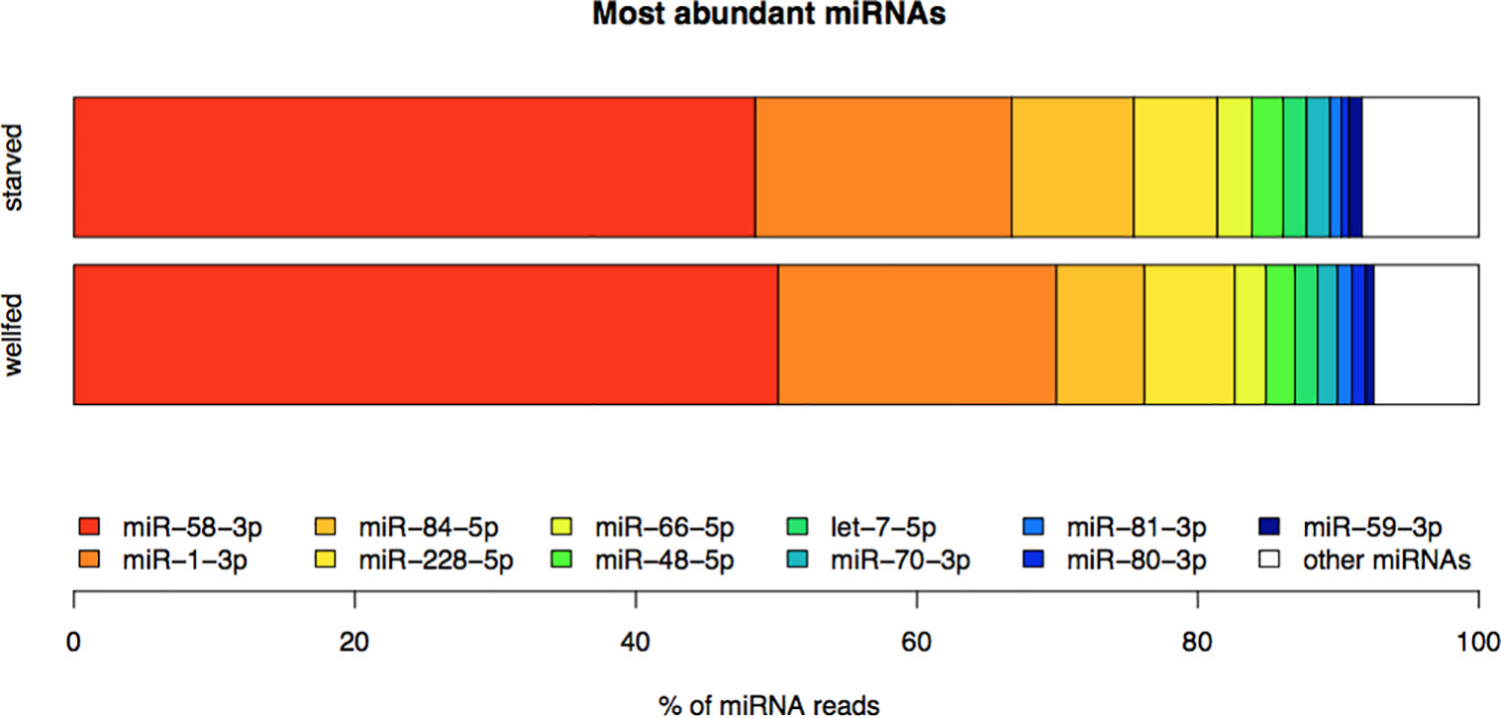
The most abundant miRNAs in each library. The most abundant miRNAs in well-fed and 12-hr starved early L4 larvae are represented as the percentage of reads that mapped to known miRNAs.

Purified RNA, enriched in the range of 20 to 30 nucleotides, obtained from well-fed early L4 larvae and early L4 larvae starved for 12 hrs, was used to construct two independent libraries. We obtained 30,270,857 and 29,656,856 total reads from the well-fed and fasted libraries, respectively. After removing adapter sequences and selecting trimmed reads between 16–28 bases, we kept 23,724,403 and 22,626,023 reads, of which the fraction that mapped to mature miRNAs was 92% and 89% for well-fed and starved early L4 larvae, respectively (see Fig 4A and 4B). By far, the most highly-expressed miRNAs were miR-58-3p and miR-1-3p, accounting for 50.1% and 19.8%, respectively, of the reads that mapped to miRNAs in well-fed larvae and 48.5% and 18.2%, under starvation (Fig 5). Neither miRNA changed in a significant manner in well fed versus 12-hr starved larvae (Fig 5). These expression levels were similar to a previous report for normally fed L4 larvae stage worms [57]. In particular, the expression of miR-58-3p was found to be in the range of 44–54% and that of miR-1-3p was between 22%-32%, when measured in embryo, L1, L2, L3, L4 and adult worms [57].

*C*. *elegans* miR-58-3p is homologous to *bantam* in *Drosophila* that has the role of scaling dendrite growth to larval growth in epithelial cells and also controls dendrite/axon regeneration in the peripheral nervous system [58,59]. In *C*. *elegans*, miR-58-3p is a member of a highly expressed family that also includes miR-80, miR-81, miR-82 and miR-1834 [60]. While miR-58-3p single mutants show no developmental, functional or viability deficits, mutants deleted for four members of the mir-58 family are deficient in body size, egg laying, locomotion and cannot form dauer larvae [60]. Several mRNA targets have been identified, including an mRNA cap-binding factor of the eIF4E family (*ife-3*), proteins involved in DNA replication, repair and recombination (*rpa-1*), chromatin remodeling (*isw-1*), chromatin binding and regulation of the RNAi mechanism (*gfl-1*), or in transcription like RNA Polymerase II (B) subunit (*rpb-7*), among others [61]. It is interesting to note that other members of the mir-58 family are also amongst the top 10 most abundant in our analysis, with miR-80-3p and miR-81-3p each contributing about 1% of the miRNA-mapping reads. Comparing the abundances of miR-58-3p to that of miR-81-3p and miR-80-3p, it is clear that the first dominates, contributing more than 90% of the reads assigned to the whole family, as reported by Kato et al. [57].

The second most abundant miRNA, miR-1-3p, functions at neuromuscular junctions and is one of the most ancient animal miRNAs, with conserved expression during musculature differentiation in bilaterians [62,63]. Mutants of miR-1-3p are resistant to levamisole-induced paralysis mediated by the increased expression of its targets *unc*-29 and *unc*-63, coding for a non-alpha and an alpha subunit of the nicotinic acetylcholine receptor, respectively. Both subunits mediate fast actions of acetylcholine and bind levamisole [62]. In *Drosophila*, the homologue of miR-1-3p is required for proper growth of larval muscle [64].

### Members of the let-7 miRNA family, as well as miR-228-5p, miR-66-5p, miR-70-3p and miR-59-3p were found to be highly expressed, but did not significantly change their expression upon starvation

The third most expressed miRNA was miR-84-5p, that together with other abundant miRNAs like miR-48-5p and let-7-5p, are members of the let-7 family, that also includes miR-7, miR-241, miR-793, miR-794 and miR-795 [65]. Let-7 was one of the first identified miRNAs, and targets the lin-41 3’UTR [66]. The name was given because of the lethal phenotype of worms with ruptured vulva that die before reaching the adult stage [67]. It was found that mutations in *let-7* cause delayed temporal fates in the last larval stages and the accumulation of *let-7* at the end of the third larval stage causes downregulation of LIN-41 protein expression, leading to the adoption of later larval and adult stages [66]. The *let-7* miRNA is also one of the most conserved miRNAs in animals, as it has been found in all bilateria [63,68]. Although miRNAs from the same family potentially regulate the same targets, masking the phenotype of individual mutants, a single let-7 mutation was sufficient to cause developmental anomalies and lethality [66].

Let-7-5p is not expressed in embryo, L1 or L2 stages; its expression augments in L3 and continues high in L4 and adult stages [57]. *Let-7* expression is regulated at both transcriptional and post-transcriptional levels. For instance, LIN-28 binds endogenous let-7 pri-miRNAs, blocking Drosha processing, leading to a reduction in mature let-7 [69]. Two transcription factors regulate *let-7* expression: HBL-1, a zinc-finger transcription factor that is homologous to *Drosophila* Hunchback that represses *let-7* expression [70] and the nuclear hormone receptor DAF-12, a regulator of dauer diapause that represses *let-7* expression in the absence of its ligand, dafachronic acid [71,72]. Several transcription factor transcripts, including *daf-12*, emerged as *let-7* targets from an RNAi screen of candidate genes that contained predicted binding sites [73]. Additionally, in *C*. *elegans*, many of the well-established *let-7* target sites, including those in *lin-41*, *daf-12*, and *hbl-1*, were detected by CLIP (cross-linking immunoprecipitation) [74]. As the 3’UTR of the small GTPase *let-60*/RAS shows multiple binding sites for *let-7* family miRNAs, the lethal phenotype of *let-7* mutants that rupture through the vulva could be caused by abnormal regulation of *let-60*/RAS [75].

Another highly expressed miRNA is miR-228-5p (Fig 5). It is conserved in *Caenorhabditis briggsae* and *Ascaris lumbricoides* [76–79], but has no assigned function yet. Similarly, the functions of other abundant miRNAs like miR-66-5p, miR-70-3p and miR-59-3p are currently unknown.

### miRNAs that changed their abundance under starvation conditions

**Fig 6:**
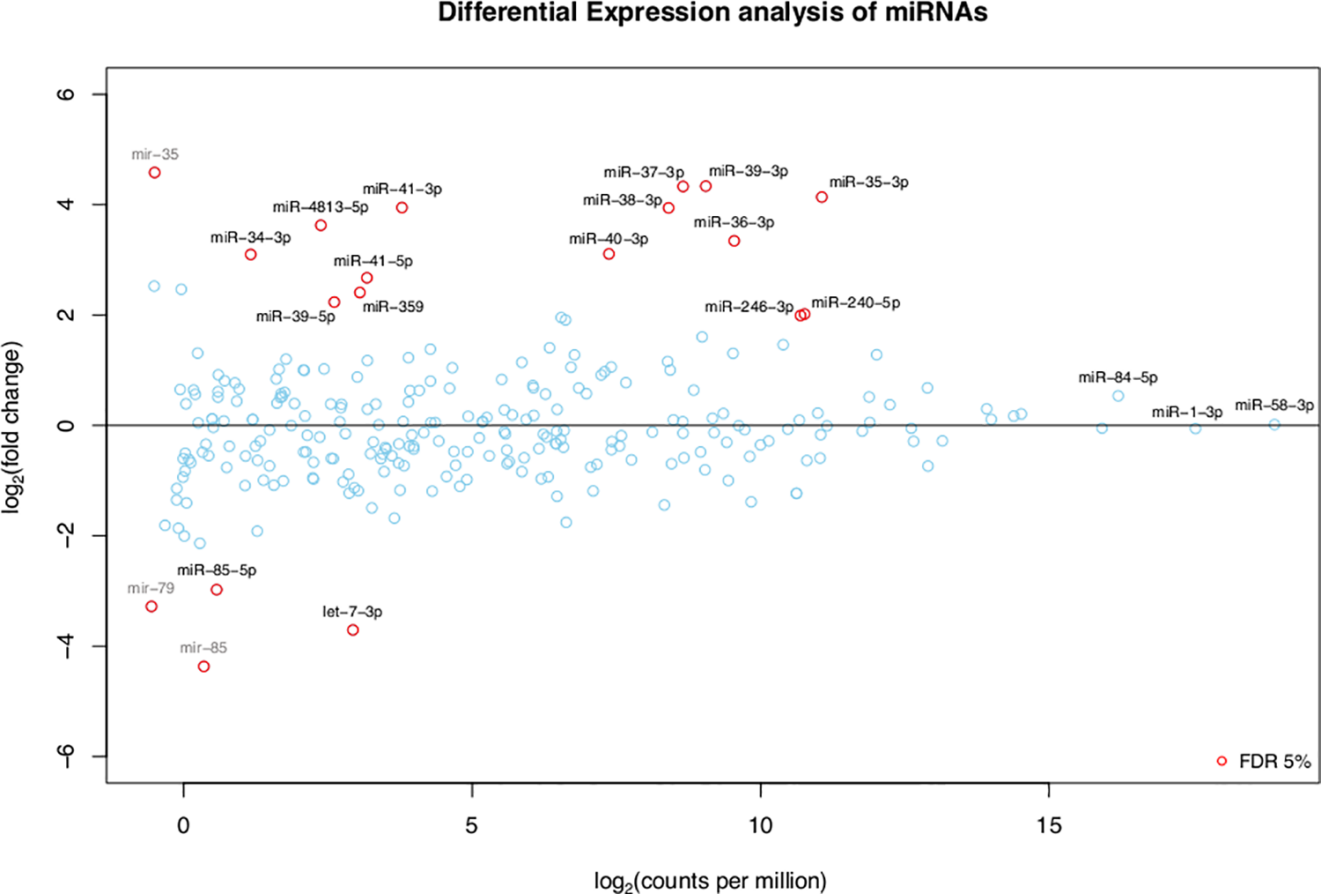
Differential expression analysis of miRNAs that changed their expression in 12-hr starved compared to well-fed early L4 larvae. MA-plot showing the absolute and relative expression of all known miRNAs with at least 1 read per million in one of the two libraries (well-fed and 12-hr starved worms). Positive (negative) log_2_ fold-changes represent miRNAs with higher (lower) expression under fasting conditions. Significantly differentially expressed miRNAs (False Discovery Rate <5%) are shown as red circles. Grey text indicates three miRNA hairpins (excluding annotated mature products) also found to be differentially expressed.

Differential expression analysis of the deep sequencing results shows that 13 miRNAs and 1 miRNA hairpin were upregulated, while 2 miRNAs and two miRNA hairpins were downregulated in 12-hr starved vs well-fed early L4 larvae (Fig 6, Table 1).

**Table 1:** Differentially expressed miRNAs and miRNA hairpins under starvation conditions.

microRNAs	Well-fed norm counts per million	12-hr starved norm counts per million	Log_2_FC	*p*-value	FDR^1^	FC^2^
cel-miR-39-3p	49.9	1008.5	4.3	6.9E-09	9.4E-07	20.2
cel-miR-37-3p	38.2	768	4.3	7.5E-09	9.4E-07	20.1
cel-miR-35-3p	230.1	4053.5	4.1	2.4E-08	2.0E-06	17.6
cel-miR-38-3p	41.3	636.1	3.9	8.4E-08	5.3E-06	15.4
cel-miR-41-3p	1.7	25.8	4.0	2.5E-07	1.3E-05	15.4
cel-let-7-3p	13.9	1.1	-3.7	1.7E-06	7.0E-05	-13.0
cel-miR-36-3p	133.3	1356.2	3.4	2.9E-06	1.0E-04	10.2
cel-miR-4813-5p	0.8	9.5	3.6	4.8E-06	1.5E-04	12.4
cel-miR-40-3p	34.3	296.4	3.1	1.2E-05	3.3E-04	8.6
cel-mir-85	2.2	0.1	-4.4	3.6E-05	9.1E-04	-20.6
cel-miR-34-3p	0.4	3.9	3.1	1.8E-04	4.1E-03	8.6
cel-miR-41-5p	2.4	15.5	2.7	2.3E-04	4.7E-03	6.4
cel-mir-35	0	1.2	4.6	4.9E-04	9.5E-03	24.0
cel-miR-359	2.6	13.9	2.4	8.2E-04	1.5E-02	5.3
cel-miR-85-5p	2.4	0.3	-3.0	1.3E-03	2.1E-02	-7.9
cel-miR-39-5p	2.1	10	2.2	1.9E-03	3.0E-02	4.7
cel-miR-240-5p	687.2	2784.7	2.0	2.9E-03	4.3E-02	4.1
cel-miR-246-3p	665.3	2646.1	2.0	3.3E-03	4.6E-02	4.0
cel-mir-79	1.0	0.1	-3.3	3.6E-03	4.8E-02	-9.7

List of differentially expressed miRNAs and miRNA hairpins deregulated during the starvation stress response in early L4 larvae of *C*. *elegans*, their expression level (counts per million reads) and fold change.

^1^ False Discovery Rate

^2^ Fold-change was calculated by dividing the higher "counts per million" by the lower, but adding the sign from the logFC to indicate direction.

As can be seen in Table 1, seven members of the miR-35 family (from miR-35-3p to miR-41-3p; [80]) were upregulated from ~6- to 20-fold under starvation conditions. This result is consistent with a previous study that found up regulation of the miR-35-41 family in a genetic model of dietary restriction in *C*. *elegans* [24]. Another miRNA found to be upregulated was miR-246-3p, that has been suggested to promote longevity, since mutants in this miRNA display a shorter lifespan and its overexpression leads to increased lifespan [81]. Although the targets of miR-246-3p have not been identified, *aakg-1* (one of the five gamma subunits of AMP kinase) and *lin-37* (involved in larval, embryo and vulval development) have been predicted as targets.

### miRNA “star” strands

We observed in our data that miR-34-3p, miR-39-5p, miR-41-5p, miR-240-5p, miR-48-13-5p were upregulated upon starvation conditions, while let-7-3p and miR-85-5p were downregulated (Fig 5, Table 1). All of them have in common that they represent the less abundant forms of the two potential miRNAs encoded in each miRNA hairpin, previously referred to as the “star” (*) strands. The miRNA star sequence is the strand complementary to the mature miRNA in the miRNA duplex. Initially, star strands were considered to be by-products of the miRNA processing steps that were rapidly degraded. But lately, star strands have been found incorporated into Argonaute complexes in *Drosophila* [82–84]. In one report, the star strands were found to interact with Drosophila AGO2, which is also associated with the siRNA pathway while the mature strands bound to AGO1, involved in translational repression [83]. Additionally, the star strands were shown to repress the expression of synthetic targets in an *in vitro* model, highlighting the capacity of star strands to act as regulatory molecules [85].

In *C*. *elegans*, star strands bind preferentially to the AGO protein RDE-1, which is required during RNA-mediated interference (RNAi) for specific removal of the passenger strand [86]. Star strands have demonstrable impact on vertebrate regulatory networks and should be considered in studies of miRNA function and their contribution to disease states. We are still lacking information about the importance of star strands in regulation, as well as the role, if any, that they play in the siRNA pathway. Star strands are predicted to have targets that are different from those of the mature strand and star strands could base pair with the mature strands and interfere with miRNA-mRNA interaction. Because an RNA molecule can base pair with other RNAs, and considering that most of the genome is transcribed, star strands may be part of a complex regulatory network in which they compete for miRNA binding and amongst each other, as has recently been postulated for competing endogenous RNAs or ceRNAs [87].

It will be interesting to discover the functions of the star strands that changed their expression under starvation conditions, whether they are bound by RDE-1 and consequently involved in the endogenous siRNA pathway, interfere with the function of their mature counterparts or have regulatory functions on their own.

### miRNA hairpins

The cleavage of pre-miRNA hairpins by Dicer generates the mature miRNA, the miRNA* (star) strand and the intervening terminal loop (loop-miR). Although miRNA* and loop-miRs were largely regarded as processing by-products of miRNA biogenesis, their regulatory role has now been demonstrated. Recently, selected loop-miRs were found enriched in Argonaute complexes in multiple drosophilids and in humans. These molecules are competent for repressing the expression of mRNAs by base pairing in standard luciferase assays [88,89]. In our analysis we were able to detect three miRNA hairpins that were differentially expressed, even though their absolute counts were quite low. This is the case of mir-35, which was upregulated, and mir-79 and mir-85, both downregulated upon starvation. It remains to be seen if these loop-miRs are incorporated into Argonaute complexes and to determine their possible regulatory role.

### Starvation-responsive miRNAs could target genes with metabolic and developmental functions

The differentially expressed miRNA sequences were used as queries to search for targets using microRNA.org [41], miRanda [42] and TargetScan [43] websites. We found several mRNAs that code for proteins involved in metabolism and development as putative targets of miRNAs that changed their expression under starvation conditions. For example, for those involved in metabolic processes we identified genes involved in the Insulin/IGF-1 signaling (IIS) such as *ins-9*, *vang-1*, *skn-1*, predicted mRNA targets of miR-39-5p, miR-41-5p, miR-240-5p, respectively (S2 Table and S1 File). As they participate in the IIS pathway, the proteins are expressed in response to nutrient availability and mutants in their coding genes show an increased lifespan. Such features could explain why L4 larvae that were starved for 12-hr showed an increased lifespan.

Other mRNAs predicted as targets of differentially expressed miRNAs are involved in lipid metabolism such as *B0301*.*1* that encodes a protein involved in lipid accumulation and could be targeted by miR-4813-5p, and *nhr-28*, that codes for a nuclear hormone receptor that functions in lipid storage and could be targeted by miR-85-5p (S2 Table and S1 File). Predicted targets that are involved in lipid metabolism could help to explain the decrease of lipids shown by worms that were starved for 12 hrs (Fig 1C). These predictions seem to be consistent with the results reported by Van Gilst et al. [90], which showed that fasting produced changes in the abundance of several mRNAs from genes involved in processes such as mitochondrial β-oxidation and synthesis of mono and polyunsaturated fatty acids, whose expression could be regulated by miRNAs.

Of the genes that are involved in development we found *gck-4* as a predicted target of miR-39-5p, and *glh-2* and *lag-1*, predicted targets of miR-240-5p (S3 Table and S1 File). The proteins coded by such mRNAs play important roles in the development of the germline, cellular proliferation, fecundity, oogenesis and growth.

### miR-240-5p, miR-246-3p, and the miR-35-41 cluster were upregulated under starvation conditions

**Fig 7:**
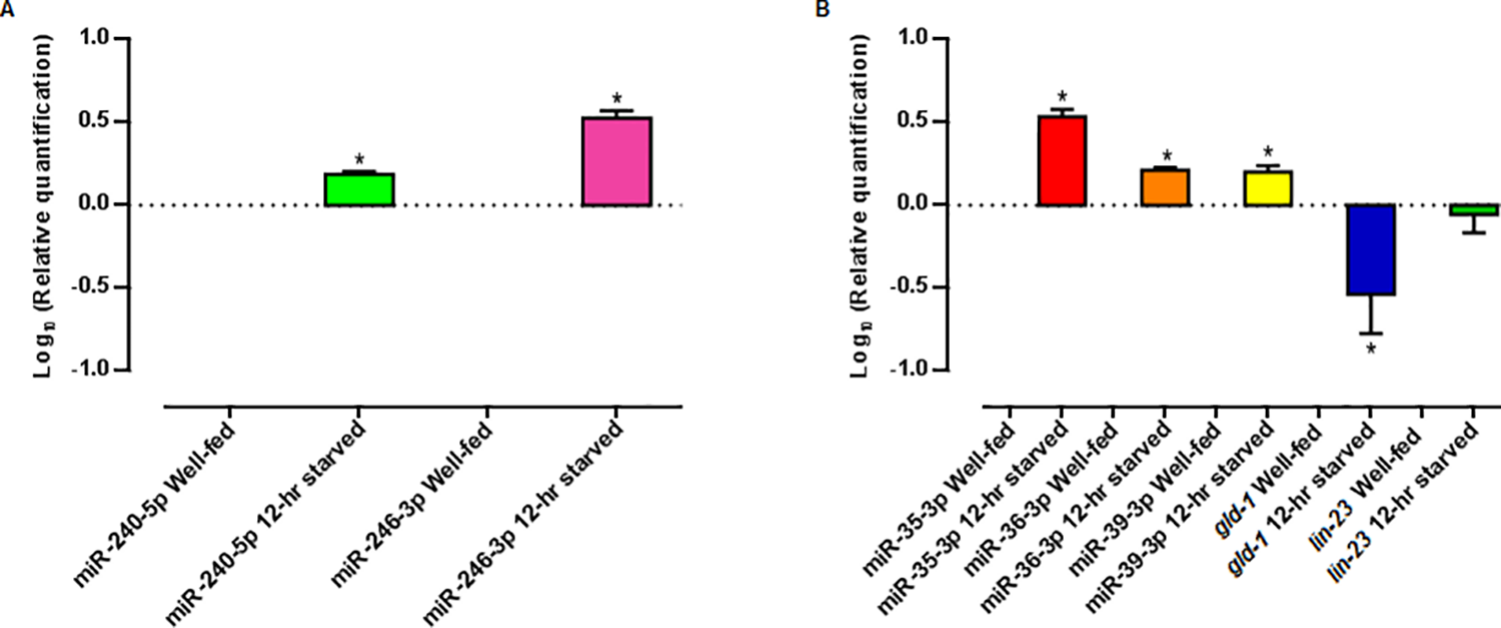
Expression of selected miRNAs and mRNAs involved in the fasting response as quantified by qRT-PCR. Relative quantification of (A) miR-240-5p, miR-246-3p, and (B) miR-35-3p, miR-36-3p, miR-39-3p, and *gld-1* and *lin-23* mRNA abundance in well-fed and 12-hr starved early L4 larvae by qRT-PCR. For miR-240-5p, miR-246-3p, miR-35-3p, miR-36-3p, miR-39-3p quantification, miR-58-3p was used as a control. For *gld-1* and *lin-23* mRNAs quantification, *β-actin* mRNA was used as a control. Error bars represent the standard error of the mean of three independent experiments. (miR-240-5p) p = 0.03, (miR-246-3p) p = 0.03, (miR-35-3p, SEM = 37.60) p = 0.03, (miR-36-3p, SEM = 14.33) p = 0.03, (miR-39-3p) p = 0.03, (*gld-1*, SEM = 20.87) p = 0.03 and (*lin-23*, SEM = 19.74) p = 0.4, calculated by the Wilcoxon Signed Rank Test.

We have shown that several miRNAs were differentially expressed in starved worms (Fig 5, Table 1). We validated the reliability of the RNA-seq profiling data by quantifying transcript abundances of selected miRNAs using qRT-PCR. We observed that the expression of miR-240-5p and miR-246-3p was upregulated in 12-hr starved worms, as seen in the RNA-seq experiments (Fig 7A). Also, we confirmed that the expression of three members of the miR-35-41 family (miR-35-3p, miR-36-3p, and miR-39-3p) was upregulated in 12-hr starved worms, consistent with the RNA-seq results in which the expression of all of the -3p forms of the miRNAs that constitute the *mir-35-41* cluster were upregulated from 6 to 20 times under starvation conditions in early L4 larvae (Fig 7B). For these experiments, we used miR-58-3p as loading control, since its expression was not significantly changed under starvation conditions.

The *mir-35* family is composed of eight miRNAs, *mir-35* to *mir-42*, all of them located on chromosome II. One cluster contains *mir-35* through *mir-41* (*mir-35-41*), while the other contains *mir-42*, *mir-43* and *mir-44*, although the last two do not belong to the *mir-35* family [80]. The *mir-35* family is conserved in worms and planaria [80,91]. In *C*. *briggsae* and *C*. *remanei* it is composed by 17 members, while *C*. *brenneri* has 27 [92]. Most embryo cells express *mir-35* members, first at the onset of gastrulation, with a peak at the onset of elongation [11,57,60]. A mutant with a deletion of all eight members of the *mir-35* family resulted in temperature-sensitive embryonic or L1 larval lethality [60]. Since the individual expression of each of the members of the *mir-35* family (but not the unrelated *mir-43* and *mir-44*) rescued the defects caused by deletion of all family members, it appears that miRNAs of the *mir-35* family act redundantly [60]. The *mir-35-41* cluster genes were also found to be expressed specifically in oogenesis and not in spermatogenesis [60]. In addition to its participation in germ cell proliferation, a key role has been ascribed to *mir-35* family members in the G1/S transition in intestinal cells, as loss of *mir-35* shows a significant decrease of nuclei numbers in both the intestine and the distal mitotic gonad [93].

Recently, two other roles have been found for the *mir-35-41* cluster. Firstly, these miRNAs are important for RNAi functions [94]. Worms lacking the *mir-35-41* cluster showed a reduced expression of *lin35*/Rb, the *C*. *elegans* homolog of the tumor suppressor Retinoblastoma gene, which is also involved in RNAi responsiveness [94]. The *lin-35*/Rb gene is a member of the synthetic multivulva B (synMuv B) family, including transcriptional repressor and chromatin remodeling genes [95]. The *mir-35-41* cluster inhibits the exogenous RNAi pathway by positively regulating the expression of LIN-35/Rb protein, although the mechanism is not known [94]. This result exemplifies the fact that miRNAs, besides regulating gene expression by direct binding to mRNAs, also have the ability to affect the activity of other small RNA pathways [94]. Secondly, the *mir-35-41* cluster miRNAs have important roles for full reproductive capacity, as a temperature sensitive mutant (a deletion of the *mir-35-41* cluster) presents reduced hermaphrodite fecundity as a result of sperm defects and somatic gonad dysfunction, and showed morphogenesis defects of male-specific mating structures [96].

In our experiments, the *mir-35-41* cluster was up-regulated in early L4 larvae subjected to a 12-hr fasting. This contrasts with the normal expression of this family in the embryo stage only. Given the functions ascribed to the members of the *mir-35* family, their up-regulation can have effects on RNAi sensitivity and reproduction. In the 12-hr starvation condition, up-regulation of miR-35-3p will negatively regulate G1/S transition in intestinal cells and will negatively regulate oogenesis [93]. This makes sense under starvation, since cell division and oogenesis are not essential, cells being focused on survival, not on making more cells or taking part in reproductive functions. Since *mir-35-41* mutants also show an increased sensitivity to endogenous RNAi [94], we can predict that starvation should cause a reduction in sensitivity to endogenous RNAi, since the expression of *mir-35-41* members are enhanced under this condition. If this is the case, it will be interesting to investigate what are the physiological consequences of having reduced sensitivity to endogenous RNAi during starvation.

### Changes in the abundance of *gld-1* and *lin-23* mRNAs during starvation, known targets of miR-35-3p

The *gld-1* and *lin-23* mRNAs have been reported to be direct targets of miR-35-3p [93]. LIN-23 (abnormal cell LINeage) is the F-box component of the SCF (Skp1-Cul1-F box) complex [97,98]. This complex shows activity of E3 ubiquitin ligase involved in the degradation of CYE-1 (cyclin E-1) [99,100] and CDC-25.1 (cell division cycle related) [101], and participates in the negative regulation of the G1/S transition. Consistent with this, a mutant in *lin-23* develops intestinal hyperplasia [101]. GLD-1 (defective in Germ Line Development) is an RNA-binding protein, member of the STAR (for signal transduction and activation of RNA metabolism) family that includes human/mouse QUAKING, SAM68, and *Drosophila* HOW [102]. The STAR proteins contain a conserved region with a maxi-KH binding domain and two conserved flanking domains (Qua1 and Qua2) [102]. The GLD family plays an important role in the determination of meiosis start, by indirectly regulating mitotic proliferation of the germline [103,104], and forms part of the regulatory network of GLP-1 (abnormal Germ Line Proliferation)/Notch signaling that promotes germ line cell divisions in the distal mitotic region of the gonad [105,106]. In the adult hermaphrodite worm, germ cells that will develop into oocytes are produced in the proliferative zone, the distal part of the gonad arm, and differentiate into oocytes as they move away [107]. Since GLD-1 is an important regulator of the start of meiosis, its expression is highly regulated at the level of mRNA stability and mRNA translation [108–110]. A mutant of GLD-1 showed a defect in oogenesis and formation of tumors in the proximal part of the gonad [102].

Taking into account the important functions of GLD-1 and LIN-23, we were interested in assessing the expression of *lin-23* and *gld-1* mRNAs in 12-hr starved L4 larvae. In the case of *lin-23* mRNA, we did not detect a significant change in its level upon starvation (Fig 7B). This could be explained if the binding of miR-35-3p to *lin-23* mRNA leads to translational repression [111,112]. In this context, it would be informative to experimentally assess if the LIN-23 protein level is affected by a 12-hr fasting.

In contrast, we found that whereas miR-35-3p expression was upregulated in 12-hr starved larvae, *gld-1* mRNA was downregulated (Fig 7B), a result that is consistent with the idea that an increase of miR-35-3p would lead to the degradation of its target *gld-1* mRNA. Such type of regulation in which the binding of a miRNA to its target mRNA leads to a destabilization of the message has been shown to be the predominant way by which miRNAs regulate gene expression in mammalian cells [113,114]. It is important to experimentally elucidate if mRNA destabilization is the mechanism used by miRNAs to regulate gene expression in *C*. *elegans* in general, and, in particular, if the down regulation of *gld-1* mRNA observed under a 12-hr fasting is mediated by a direct binding of miR-35-3p.

## Role of microRNAs in Reproduction and Lifespan

The relationship between metabolism, reproduction and lifespan has been studied and involves changes in the activities of signaling pathways like Insulin/IGF and TOR [115]. Different mutations in the Insulin/IGF signaling pathway in genes like *daf-2* (Insulin/IGF receptor ortholog) and *daf-16* (a FOXO like transcription factor) increase lifespan by affecting genes involved in a diversity of process like metabolism, stress response, innate immunity, signaling, germ line development, among others [44]. Dietary restriction is also known to increase lifespan in several species and intervenes in the reproductive function by regulating DAF-9/CYP450, steroid hormone Δ^7^-dafachronic acid/DAF-12, NHR-8/NHR and *let-363*/TOR [52]. Interestingly, in such regulation the participation of miRNAs like miR-71, miR-84 and miR-241 is very important as these miRNAs down regulate the expression of AKT-1 and LIN-14, resulting in the activation of DAF-16 [116]. With this in mind, we can assert that metabolism, reproduction and lifespan are coordinately regulated by different molecules. We observed that the lack of food produced a change in the expression of different miRNAs that could be important for the regulation of these processes.

In our results, early L4 larvae subjected to a 12-hr starvation showed morphological alterations in the gonad tissue and a lowered brood size, which can be in part explained by the observed down regulation of *gld-1* mRNA, probably as a result of the increased abundance of one of its regulators, the miR-35-41 family. So, low availability of nutrients in early stages of development impacts the germ cell pool and indirectly lifespan, in a mechanism that is mediated by miRNAs. In line with this concept is the low fertility rate, sperm defects and dysfunctional development of male structures observed in mutants of the *mir-35-41* family [96]. Although there is still much to be discovered about the relationship between metabolism, reproduction and lifespan, the regulation provided by miRNAs could help us understand the interaction between these processes. Additionally, the knowledge of how microRNAs coordinate gene expression in response to starvation could help us to further identify the molecular mechanisms involved in aging, obesity and cancer.

## Supporting Information

S1 File References for S2 and S3 Tables. (PDF)

S1 Table List of primers. Primers used for Reverse Transcription and qPCR. The Universal ProbeLibrary Probe #21 binding sites are highlighted in red.
(PDF)

S2 Table Predicted miRNA targets with metabolic functions. Targets with known metabolic functions predicted for differentially expressed miRNAs.
(PDF)

S3 Table Predicted miRNA targets with developmental functions. Targets with known developmental functions predicted for differentially expressed miRNAs.
(PDF)

## References

[1] Levine B, Yuan J (2005) Autophagy in cell death: an innocent convict? J Clin Invest 115: 2679–2688. 16200202 10.1172/JCI26390 PMC1236698

[2] Lum JJ, DeBerardinis RJ, Thompson CB (2005) Autophagy in metazoans: cell survival in the land of plenty. Nat Rev Mol Cell Biol 6: 439–448. 15928708 10.1038/nrm1660

[3] Leung AK, Sharp PA (2010) MicroRNA functions in stress responses. Mol Cell 40: 205–215. 10.1016/j.molcel.2010.09.027 20965416 PMC2996264

[4] Carthew RW, Sontheimer EJ (2009) Origins and Mechanisms of miRNAs and siRNAs. Cell 136: 642–655. 10.1016/j.cell.2009.01.035 19239886 PMC2675692

[5] Friedman RC, Farh KK, Burge CB, Bartel DP (2009) Most mammalian mRNAs are conserved targets of microRNAs. Genome Res 19: 92–105. 10.1101/gr.082701.108 18955434 PMC2612969

[6] Kozomara A, Griffiths-Jones S (2014) miRBase: annotating high confidence microRNAs using deep sequencing data. Nucleic Acids Res 42: D68–73. 10.1093/nar/gkt1181 24275495 PMC3965103

[7] Londin E, Loher P, Telonis AG, Quann K, Clark P, et al (2015) Analysis of 13 cell types reveals evidence for the expression of numerous novel primate- and tissue-specific microRNAs. Proc Natl Acad Sci U S A 112: E1106–1115. 10.1073/pnas.1420955112 25713380 PMC4364231

[8] Leung AK, Sharp PA (2007) microRNAs: a safeguard against turmoil? Cell 130: 581–585. 17719533 10.1016/j.cell.2007.08.010

[9] Bushati N, Cohen SM (2007) microRNA functions. Annu Rev Cell Dev Biol 23: 175–205. 17506695 10.1146/annurev.cellbio.23.090506.123406

[10] Leaman D, Chen PY, Fak J, Yalcin A, Pearce M, et al (2005) Antisense-mediated depletion reveals essential and specific functions of microRNAs in Drosophila development. Cell 121: 1097–1108. 15989958 10.1016/j.cell.2005.04.016

[11] Miska EA, Alvarez-Saavedra E, Abbott AL, Lau NC, Hellman AB, et al (2007) Most Caenorhabditis elegans microRNAs are individually not essential for development or viability. PLoS Genet 3: e215 18085825 10.1371/journal.pgen.0030215 PMC2134938

[12] Li X, Cassidy JJ, Reinke CA, Fischboeck S, Carthew RW (2009) A microRNA imparts robustness against environmental fluctuation during development. Cell 137: 273–282. 10.1016/j.cell.2009.01.058 19379693 PMC2674871

[13] Vanfleteren JR, Braeckman BP (1999) Mechanisms of life span determination in Caenorhabditis elegans. Neurobiol Aging 20: 487–502. 10638522 10.1016/s0197-4580(99)00087-1

[14] Hope IA (2002) C. elegans: a practical approach Oxford; New York: Oxford University Press xxi, 281 p. p.

[15] Johnson TE, Mitchell DH, Kline S, Kemal R, Foy J (1984) Arresting development arrests aging in the nematode Caenorhabditis elegans. Mech Ageing Dev 28: 23–40. 6542614 10.1016/0047-6374(84)90150-7

[16] Paradis S, Ailion M, Toker A, Thomas JH, Ruvkun G (1999) A PDK1 homolog is necessary and sufficient to transduce AGE-1 PI3 kinase signals that regulate diapause in Caenorhabditis elegans. Genes Dev 13: 1438–1452. 10364160 10.1101/gad.13.11.1438 PMC316759

[17] Than M, Han M (2013) Functional analysis of the miRNA-mRNA interaction network in C. elegans. Worm 2: e26894 10.4161/worm.26894 24744982 PMC3917963

[18] Garcia-Segura L, Perez-Andrade M, Miranda-Rios J (2013) The emerging role of MicroRNAs in the regulation of gene expression by nutrients. J Nutrigenet Nutrigenomics 6: 16–31. 10.1159/000345826 23445777

[19] Zhang X, Zabinsky R, Teng Y, Cui M, Han M (2011) microRNAs play critical roles in the survival and recovery of Caenorhabditis elegans from starvation-induced L1 diapause. Proc Natl Acad Sci U S A 108: 17997–18002. 10.1073/pnas.1105982108 22011579 PMC3207661

[20] Karp X, Ambros V (2011) The developmental timing regulator HBL-1 modulates the dauer formation decision in Caenorhabditis elegans. Genetics 187: 345–353. 10.1534/genetics.110.123992 20980238 PMC3018311

[21] Pincus Z, Smith-Vikos T, Slack FJ (2011) MicroRNA predictors of longevity in Caenorhabditis elegans. PLoS Genet 7: e1002306 10.1371/journal.pgen.1002306 21980307 PMC3183074

[22] Boulias K, Horvitz HR (2012) The C. elegans microRNA mir-71 acts in neurons to promote germline-mediated longevity through regulation of DAF-16/FOXO. Cell Metab 15: 439–450. 10.1016/j.cmet.2012.02.014 22482727 PMC3344382

[23] Smith-Vikos T, de Lencastre A, Inukai S, Shlomchik M, Holtrup B, et al (2014) MicroRNAs mediate dietary-restriction-induced longevity through PHA-4/FOXA and SKN-1/Nrf transcription factors. Curr Biol 24: 2238–2246. 10.1016/j.cub.2014.08.013 25242029 PMC4208828

[24] Pandit A, Jain V, Kumar N, Mukhopadhyay A (2014) PHA-4/FOXA-regulated microRNA feed forward loops during Caenorhabditis elegans dietary restriction. Aging (Albany NY) 6: 835–855. 25504288 10.18632/aging.100697 PMC4247386

[25] Karp X, Hammell M, Ow MC, Ambros V (2011) Effect of life history on microRNA expression during C. elegans development. RNA 17: 639–651. 10.1261/rna.2310111 21343388 PMC3062175

[26] Lim LP, Lau NC, Garrett-Engele P, Grimson A, Schelter JM, et al (2005) Microarray analysis shows that some microRNAs downregulate large numbers of target mRNAs. Nature 433: 769–773. 15685193 10.1038/nature03315

[27] Kato M, Paranjape T, Muller RU, Nallur S, Gillespie E, et al (2009) The mir-34 microRNA is required for the DNA damage response in vivo in C. elegans and in vitro in human breast cancer cells. Oncogene 28: 2419–2424. 10.1038/onc.2009.106 19421141 PMC2941141

[28] Kato M, Slack FJ (2013) Ageing and the small, non-coding RNA world. Ageing Res Rev 12: 429–435. 10.1016/j.arr.2012.03.012 22504407 PMC3405179

[29] Gong G, Sha Z, Chen S, Li C, Yan H, et al (2015) Expression profiling analysis of the microRNA response of Cynoglossus semilaevis to Vibrio anguillarum and other stimuli. Mar Biotechnol (NY) 17: 338–352. 25715708 10.1007/s10126-015-9623-2

[30] Wen J, Leucci E, Vendramin R, Kauppinen S, Lund AH, et al (2015) Transcriptome dynamics of the microRNA inhibition response. Nucleic Acids Res 43: 6207–6221. 10.1093/nar/gkv603 26089393 PMC4513874

[31] Lukowski SW, Fish RJ, Martin-Levilain J, Gonelle-Gispert C, Buhler LH, et al (2015) Integrated analysis of mRNA and miRNA expression in response to interleukin-6 in hepatocytes. Genomics 106: 107–115. 10.1016/j.ygeno.2015.05.001 25979460

[32] Larance M, Pourkarimi E, Wang B, Murillo AB, Kent R, et al (2015) Global proteomics analysis of the response to starvation in C. elegans. Mol Cell Proteomics. 10.1074/mcp.M114.044289 PMC4587315 25963834

[33] Wood WB (1988) The Nematode Caenorhabditis elegans Cold Spring Harbor, N.Y.: Cold Spring Harbor Laboratory xiii, 667 p. p.

[34] Yook K, Harris TW, Bieri T, Cabunoc A, Chan J, et al (2012) WormBase 2012: more genomes, more data, new website. Nucleic Acids Res 40: D735–741. 10.1093/nar/gkr954 22067452 PMC3245152

[35] Langmead B, Trapnell C, Pop M, Salzberg SL (2009) Ultrafast and memory-efficient alignment of short DNA sequences to the human genome. Genome Biol 10: R25 10.1186/gb-2009-10-3-r25 19261174 PMC2690996

[36] Davis MP, van Dongen S, Abreu-Goodger C, Bartonicek N, Enright AJ (2013) Kraken: a set of tools for quality control and analysis of high-throughput sequence data. Methods 63: 41–49. 10.1016/j.ymeth.2013.06.027 23816787 PMC3991327

[37] Gentleman RC, Carey VJ, Bates DM, Bolstad B, Dettling M, et al (2004) Bioconductor: open software development for computational biology and bioinformatics. Genome Biol 5: R80 15461798 10.1186/gb-2004-5-10-r80 PMC545600

[38] Hackenberg M, Sturm M, Langenberger D, Falcon-Perez JM, Aransay AM (2009) miRanalyzer: a microRNA detection and analysis tool for next-generation sequencing experiments. Nucleic Acids Res 37: W68–76. 10.1093/nar/gkp347 19433510 PMC2703919

[39] Robinson MD, McCarthy DJ, Smyth GK (2010) edgeR: a Bioconductor package for differential expression analysis of digital gene expression data. Bioinformatics 26: 139–140. 10.1093/bioinformatics/btp616 19910308 PMC2796818

[40] Benjamini Y, Hochberg Y (1995) Controlling the False Discovery Rate—a Practical and Powerful Approach to Multiple Testing. Journal of the Royal Statistical Society Series B-Methodological 57: 289–300.

[41] Betel D, Wilson M, Gabow A, Marks DS, Sander C (2008) The microRNA.org resource: targets and expression. Nucleic Acids Research 36: D149–D153. 18158296 10.1093/nar/gkm995 PMC2238905

[42] Miranda KC, Huynh T, Tay Y, Ang YS, Tam WL, et al (2006) A pattern-based method for the identification of MicroRNA binding sites and their corresponding heteroduplexes. Cell 126: 1203–1217. 16990141 10.1016/j.cell.2006.07.031

[43] Jan CH, Friedman RC, Ruby JG, Bartel DP (2011) Formation, regulation and evolution of Caenorhabditis elegans 3'UTRs. Nature 469: 97–101. 10.1038/nature09616 21085120 PMC3057491

[44] Zhang P, Judy M, Lee SJ, Kenyon C (2013) Direct and indirect gene regulation by a life-extending FOXO protein in C. elegans: roles for GATA factors and lipid gene regulators. Cell Metab 17: 85–100. 10.1016/j.cmet.2012.12.013 23312285 PMC3969420

[45] Chen C, Ridzon DA, Broomer AJ, Zhou Z, Lee DH, et al (2005) Real-time quantification of microRNAs by stem-loop RT-PCR. Nucleic Acids Res 33: e179 16314309 10.1093/nar/gni178 PMC1292995

[46] Varkonyi-Gasic E, Wu R, Wood M, Walton EF, Hellens RP (2007) Protocol: a highly sensitive RT-PCR method for detection and quantification of microRNAs. Plant Methods 3: 12 17931426 10.1186/1746-4811-3-12 PMC2225395

[47] Pfaffl MW (2001) A new mathematical model for relative quantification in real-time RT-PCR. Nucleic Acids Res 29: e45 11328886 10.1093/nar/29.9.e45 PMC55695

[48] Srinivasan S (2015) Regulation of body fat in Caenorhabditis elegans. Annu Rev Physiol 77: 161–178. 10.1146/annurev-physiol-021014-071704 25340962 PMC4766980

[49] Avery L (1993) The genetics of feeding in Caenorhabditis elegans. Genetics 133: 897–917. 8462849 10.1093/genetics/133.4.897 PMC1205408

[50] Holliday R (1989) Food, reproduction and longevity: is the extended lifespan of calorie-restricted animals an evolutionary adaptation? Bioessays 10: 125–127. 2730632 10.1002/bies.950100408

[51] Klass MR (1977) Aging in the nematode Caenorhabditis elegans: major biological and environmental factors influencing life span. Mech Ageing Dev 6: 413–429. 926867 10.1016/0047-6374(77)90043-4

[52] Thondamal M, Witting M, Schmitt-Kopplin P, Aguilaniu H (2014) Steroid hormone signalling links reproduction to lifespan in dietary-restricted Caenorhabditis elegans. Nat Commun 5: 4879 10.1038/ncomms5879 25209682

[53] Angelo G, Van Gilst MR (2009) Starvation protects germline stem cells and extends reproductive longevity in C. elegans. Science 326: 954–958. 10.1126/science.1178343 19713489

[54] Seidel HS, Kimble J (2011) The oogenic germline starvation response in C. elegans. PLoS One 6: e28074 10.1371/journal.pone.0028074 22164230 PMC3229504

[55] Roth LW, Polotsky AJ (2012) Can we live longer by eating less? A review of caloric restriction and longevity. Maturitas 71: 315–319. 10.1016/j.maturitas.2011.12.017 22281163

[56] Kaeberlein TL, Smith ED, Tsuchiya M, Welton KL, Thomas JH, et al (2006) Lifespan extension in Caenorhabditis elegans by complete removal of food. Aging Cell 5: 487–494. 17081160 10.1111/j.1474-9726.2006.00238.x

[57] Kato M, de Lencastre A, Pincus Z, Slack FJ (2009) Dynamic expression of small non-coding RNAs, including novel microRNAs and piRNAs/21U-RNAs, during Caenorhabditis elegans development. Genome Biol 10: R54 10.1186/gb-2009-10-5-r54 19460142 PMC2718520

[58] Parrish JZ, Xu P, Kim CC, Jan LY, Jan YN (2009) The microRNA bantam functions in epithelial cells to regulate scaling growth of dendrite arbors in drosophila sensory neurons. Neuron 63: 788–802. 10.1016/j.neuron.2009.08.006 19778508 PMC2772869

[59] Song Y, Ori-McKenney KM, Zheng Y, Han C, Jan LY, et al (2012) Regeneration of Drosophila sensory neuron axons and dendrites is regulated by the Akt pathway involving Pten and microRNA bantam. Genes Dev 26: 1612–1625. 10.1101/gad.193243.112 22759636 PMC3404388

[60] Alvarez-Saavedra E, Horvitz HR (2010) Many families of C. elegans microRNAs are not essential for development or viability. Curr Biol 20: 367–373. 10.1016/j.cub.2009.12.051 20096582 PMC2844791

[61] Jovanovic M, Reiter L, Picotti P, Lange V, Bogan E, et al (2010) A quantitative targeted proteomics approach to validate predicted microRNA targets in C. elegans. Nat Methods 7: 837–842. 10.1038/nmeth.1504 20835247 PMC3444237

[62] Simon DJ, Madison JM, Conery AL, Thompson-Peer KL, Soskis M, et al (2008) The microRNA miR-1 regulates a MEF-2-dependent retrograde signal at neuromuscular junctions. Cell 133: 903–915. 10.1016/j.cell.2008.04.035 18510933 PMC2553566

[63] Christodoulou F, Raible F, Tomer R, Simakov O, Trachana K, et al (2010) Ancient animal microRNAs and the evolution of tissue identity. Nature 463: 1084–1088. 10.1038/nature08744 20118916 PMC2981144

[64] Sokol NS, Ambros V (2005) Mesodermally expressed Drosophila microRNA-1 is regulated by Twist and is required in muscles during larval growth. Genes Dev 19: 2343–2354. 16166373 10.1101/gad.1356105 PMC1240043

[65] Mondol V, Pasquinelli AE (2012) Let's make it happen: the role of let-7 microRNA in development. Curr Top Dev Biol 99: 1–30. 10.1016/B978-0-12-387038-4.00001-X 22365733

[66] Reinhart BJ, Slack FJ, Basson M, Pasquinelli AE, Bettinger JC, et al (2000) The 21-nucleotide let-7 RNA regulates developmental timing in Caenorhabditis elegans. Nature 403: 901–906. 10706289 10.1038/35002607

[67] Meneely PM, Herman RK (1979) Lethals, steriles and deficiencies in a region of the X chromosome of Caenorhabditis elegans. Genetics 92: 99–115. 574105 10.1093/genetics/92.1.99 PMC1213963

[68] Pasquinelli AE, Reinhart BJ, Slack F, Martindale MQ, Kuroda MI, et al (2000) Conservation of the sequence and temporal expression of let-7 heterochronic regulatory RNA. Nature 408: 86–89. 11081512 10.1038/35040556

[69] Van Wynsberghe PM, Kai ZS, Massirer KB, Burton VH, Yeo GW, et al (2011) LIN-28 co-transcriptionally binds primary let-7 to regulate miRNA maturation in Caenorhabditis elegans. Nat Struct Mol Biol 18: 302–308. 10.1038/nsmb.1986 21297634 PMC3077891

[70] Roush SF, Slack FJ (2009) Transcription of the C. elegans let-7 microRNA is temporally regulated by one of its targets, hbl-1. Dev Biol 334: 523–534. 10.1016/j.ydbio.2009.07.012 19627983 PMC2753757

[71] Hammell CM, Karp X, Ambros V (2009) A feedback circuit involving let-7-family miRNAs and DAF-12 integrates environmental signals and developmental timing in Caenorhabditis elegans. Proc Natl Acad Sci U S A 106: 18668–18673. 10.1073/pnas.0908131106 19828440 PMC2774035

[72] Motola DL, Cummins CL, Rottiers V, Sharma KK, Li T, et al (2006) Identification of ligands for DAF-12 that govern dauer formation and reproduction in C. elegans. Cell 124: 1209–1223. 16529801 10.1016/j.cell.2006.01.037

[73] Grosshans H, Johnson T, Reinert KL, Gerstein M, Slack FJ (2005) The temporal patterning microRNA let-7 regulates several transcription factors at the larval to adult transition in C. elegans. Dev Cell 8: 321–330. 15737928 10.1016/j.devcel.2004.12.019

[74] Zisoulis DG, Yeo GW, Pasquinelli AE (2011) Comprehensive identification of miRNA target sites in live animals. Methods Mol Biol 732: 169–185. 10.1007/978-1-61779-083-6_13 21431713

[75] Johnson SM, Grosshans H, Shingara J, Byrom M, Jarvis R, et al (2005) RAS is regulated by the let-7 microRNA family. Cell 120: 635–647. 15766527 10.1016/j.cell.2005.01.014

[76] Lim LP, Lau NC, Weinstein EG, Abdelhakim A, Yekta S, et al (2003) The microRNAs of Caenorhabditis elegans. Genes Dev 17: 991–1008. 12672692 10.1101/gad.1074403 PMC196042

[77] Ambros V, Lee RC, Lavanway A, Williams PT, Jewell D (2003) MicroRNAs and other tiny endogenous RNAs in C. elegans. Curr Biol 13: 807–818. 12747828 10.1016/s0960-9822(03)00287-2

[78] Grad Y, Aach J, Hayes GD, Reinhart BJ, Church GM, et al (2003) Computational and experimental identification of C. elegans microRNAs. Mol Cell 11: 1253–1263. 12769849 10.1016/s1097-2765(03)00153-9

[79] Wang J, Czech B, Crunk A, Wallace A, Mitreva M, et al (2011) Deep small RNA sequencing from the nematode Ascaris reveals conservation, functional diversification, and novel developmental profiles. Genome Res 21: 1462–1477. 10.1101/gr.121426.111 21685128 PMC3166831

[80] Lau NC, Lim LP, Weinstein EG, Bartel DP (2001) An abundant class of tiny RNAs with probable regulatory roles in Caenorhabditis elegans. Science 294: 858–862. 11679671 10.1126/science.1065062

[81] de Lencastre A, Pincus Z, Zhou K, Kato M, Lee SS, et al (2010) MicroRNAs both promote and antagonize longevity in C. elegans. Curr Biol 20: 2159–2168. 10.1016/j.cub.2010.11.015 21129974 PMC3023310

[82] Czech B, Zhou R, Erlich Y, Brennecke J, Binari R, et al (2009) Hierarchical rules for Argonaute loading in Drosophila. Mol Cell 36: 445–456. 10.1016/j.molcel.2009.09.028 19917252 PMC2795325

[83] Okamura K, Liu N, Lai EC (2009) Distinct mechanisms for microRNA strand selection by Drosophila Argonautes. Mol Cell 36: 431–444. 10.1016/j.molcel.2009.09.027 19917251 PMC2785079

[84] Ghildiyal M, Xu J, Seitz H, Weng Z, Zamore PD (2010) Sorting of Drosophila small silencing RNAs partitions microRNA* strands into the RNA interference pathway. RNA 16: 43–56. 10.1261/rna.1972910 19917635 PMC2802036

[85] Yang JS, Phillips MD, Betel D, Mu P, Ventura A, et al (2011) Widespread regulatory activity of vertebrate microRNA* species. RNA 17: 312–326. 10.1261/rna.2537911 21177881 PMC3022280

[86] Kato M, Chen X, Inukai S, Zhao H, Slack FJ (2011) Age-associated changes in expression of small, noncoding RNAs, including microRNAs, in C. elegans. RNA 17: 1804–1820. 10.1261/rna.2714411 21810936 PMC3185914

[87] Salmena L, Poliseno L, Tay Y, Kats L, Pandolfi PP (2011) A ceRNA hypothesis: the Rosetta Stone of a hidden RNA language? Cell 146: 353–358. 10.1016/j.cell.2011.07.014 21802130 PMC3235919

[88] Okamura K, Ladewig E, Zhou L, Lai EC (2013) Functional small RNAs are generated from select miRNA hairpin loops in flies and mammals. Genes Dev 27: 778–792. 10.1101/gad.211698.112 23535236 PMC3639418

[89] Winter J, Link S, Witzigmann D, Hildenbrand C, Previti C, et al (2013) Loop-miRs: active microRNAs generated from single-stranded loop regions. Nucleic Acids Res 41: 5503–5512. 10.1093/nar/gkt251 23580554 PMC3664828

[90] Van Gilst MR, Hadjivassiliou H, Yamamoto KR (2005) A Caenorhabditis elegans nutrient response system partially dependent on nuclear receptor NHR-49. Proc Natl Acad Sci U S A 102: 13496–13501. 16157872 10.1073/pnas.0506234102 PMC1201344

[91] Lu YC, Smielewska M, Palakodeti D, Lovci MT, Aigner S, et al (2009) Deep sequencing identifies new and regulated microRNAs in Schmidtea mediterranea. RNA 15: 1483–1491. 10.1261/rna.1702009 19553344 PMC2714757

[92] Shi Z, Hayes G, Ruvkun G (2013) Dual regulation of the lin-14 target mRNA by the lin-4 miRNA. PLoS One 8: e75475 10.1371/journal.pone.0075475 24058689 PMC3772890

[93] Liu M, Liu P, Zhang L, Cai Q, Gao G, et al (2011) mir-35 is involved in intestine cell G1/S transition and germ cell proliferation in C. elegans. Cell Res 21: 1605–1618. 10.1038/cr.2011.102 21691303 PMC3364723

[94] Massirer KB, Perez SG, Mondol V, Pasquinelli AE (2012) The miR-35-41 family of microRNAs regulates RNAi sensitivity in Caenorhabditis elegans. PLoS Genet 8: e1002536 10.1371/journal.pgen.1002536 22412382 PMC3297572

[95] Lu X, Horvitz HR (1998) lin-35 and lin-53, two genes that antagonize a C. elegans Ras pathway, encode proteins similar to Rb and its binding protein RbAp48. Cell 95: 981–991. 9875852 10.1016/s0092-8674(00)81722-5

[96] McJunkin K, Ambros V (2014) The embryonic mir-35 family of microRNAs promotes multiple aspects of fecundity in Caenorhabditis elegans. G3 (Bethesda) 4: 1747–1754. 25053708 10.1534/g3.114.011973 PMC4169167

[97] Kipreos ET, Lander LE, Wing JP, He WW, Hedgecock EM (1996) cul-1 is required for cell cycle exit in C. elegans and identifies a novel gene family. Cell 85: 829–839. 8681378 10.1016/s0092-8674(00)81267-2

[98] Kipreos ET, Gohel SP, Hedgecock EM (2000) The C. elegans F-box/WD-repeat protein LIN-23 functions to limit cell division during development. Development 127: 5071–5082. 11060233 10.1242/dev.127.23.5071

[99] Dealy MJ, Nguyen KV, Lo J, Gstaiger M, Krek W, et al (1999) Loss of Cul1 results in early embryonic lethality and dysregulation of cyclin E. Nat Genet 23: 245–248. 10508527 10.1038/13886

[100] Wang Y, Penfold S, Tang X, Hattori N, Riley P, et al (1999) Deletion of the Cul1 gene in mice causes arrest in early embryogenesis and accumulation of cyclin E. Curr Biol 9: 1191–1194. 10531039 10.1016/S0960-9822(00)80024-X

[101] Hebeisen M, Roy R (2008) CDC-25.1 stability is regulated by distinct domains to restrict cell division during embryogenesis in C. elegans. Development 135: 1259–1269. 10.1242/dev.014969 18287204

[102] Lee MH, Schedl T (2010) C. elegans star proteins, GLD-1 and ASD-2, regulate specific RNA targets to control development. Adv Exp Med Biol 693: 106–122. 21189689 10.1007/978-1-4419-7005-3_8

[103] Francis R, Barton MK, Kimble J, Schedl T (1995) gld-1, a tumor suppressor gene required for oocyte development in Caenorhabditis elegans. Genetics 139: 579–606. 7713419 10.1093/genetics/139.2.579 PMC1206368

[104] Jones AR, Francis R, Schedl T (1996) GLD-1, a cytoplasmic protein essential for oocyte differentiation, shows stage- and sex-specific expression during Caenorhabditis elegans germline development. Dev Biol 180: 165–183. 8948583 10.1006/dbio.1996.0293

[105] Austin J, Kimble J (1987) glp-1 is required in the germ line for regulation of the decision between mitosis and meiosis in C. elegans. Cell 51: 589–599. 3677168 10.1016/0092-8674(87)90128-0

[106] Berry LW, Westlund B, Schedl T (1997) Germ-line tumor formation caused by activation of glp-1, a Caenorhabditis elegans member of the Notch family of receptors. Development 124: 925–936. 9043073 10.1242/dev.124.4.925

[107] Lehmann R (2012) Germline stem cells: origin and destiny. Cell Stem Cell 10: 729–739. 10.1016/j.stem.2012.05.016 22704513 PMC3750984

[108] Schmid M, Kuchler B, Eckmann CR (2009) Two conserved regulatory cytoplasmic poly(A) polymerases, GLD-4 and GLD-2, regulate meiotic progression in C. elegans. Genes Dev 23: 824–836. 10.1101/gad.494009 19339688 PMC2666339

[109] Suh N, Crittenden SL, Goldstrohm A, Hook B, Thompson B, et al (2009) FBF and its dual control of gld-1 expression in the Caenorhabditis elegans germline. Genetics 181: 1249–1260. 10.1534/genetics.108.099440 19221201 PMC2666496

[110] Minasaki R, Rudel D, Eckmann CR (2014) Increased sensitivity and accuracy of a single-stranded DNA splint-mediated ligation assay (sPAT) reveals poly(A) tail length dynamics of developmentally regulated mRNAs. RNA Biol 11: 111–123. 10.4161/rna.27992 24526206 PMC3973730

[111] Wightman B, Ha I, Ruvkun G (1993) Posttranscriptional regulation of the heterochronic gene lin-14 by lin-4 mediates temporal pattern formation in C. elegans. Cell 75: 855–862. 8252622 10.1016/0092-8674(93)90530-4

[112] Olsen PH, Ambros V (1999) The lin-4 regulatory RNA controls developmental timing in Caenorhabditis elegans by blocking LIN-14 protein synthesis after the initiation of translation. Dev Biol 216: 671–680. 10642801 10.1006/dbio.1999.9523

[113] Guo H, Ingolia NT, Weissman JS, Bartel DP (2010) Mammalian microRNAs predominantly act to decrease target mRNA levels. Nature 466: 835–840. 10.1038/nature09267 20703300 PMC2990499

[114] Eichhorn SW, Guo H, McGeary SE, Rodriguez-Mias RA, Shin C, et al (2014) mRNA destabilization is the dominant effect of mammalian microRNAs by the time substantial repression ensues. Mol Cell 56: 104–115. 10.1016/j.molcel.2014.08.028 25263593 PMC4292926

[115] Hansen M, Flatt T, Aguilaniu H (2013) Reproduction, fat metabolism, and life span: what is the connection? Cell Metab 17: 10–19. 10.1016/j.cmet.2012.12.003 23312280 PMC3567776

[116] Shen Y, Wollam J, Magner D, Karalay O, Antebi A (2012) A steroid receptor-microRNA switch regulates life span in response to signals from the gonad. Science 338: 1472–1476. 10.1126/science.1228967 23239738 PMC3909774

