# Supplementary material for: High-Throughput Profiling of Caenorhabditis elegans Starvation-Responsive microRNAs
Source: PLoS One. 2015 Nov 10;10(11):e0142262. doi: 10.1371/journal.pone.0142262 (PMC4640506; doi:10.1371/journal.pone.0142262)
Supplement: S1 File — (PDF) [file pone.0142262.s001.pdf]

S1 File. References for S2 and S3 Tables.

1. Hung WL, Hwang C, Gao S, Liao EH, Chitturi J, Wang Y, et al. Attenuation of insulin signalling contributes to FSN-1-mediated regulation of synapse development. The EMBO Journal. 2013;32: 1745–1760.

2. Gal TZ, Solomon A, Glazer I, Koltai H. Alterations in the levels of glycogen and glycogen synthase transcripts during desiccation in the insect-killing nematode steinernema feltiae is-6. J. Parasitol. 2001;87: 725–732.

3. Ashrafi K, Chang FY, Watts JL, Fraser AG, Kamath RS, Ahringer J, et al. Genome-wide RNAi analysis of *Caenorhabditis elegans* fat regulatory genes. Nature. 2003;421: 268-72.

4. Li H, Ren C, Shi J, Hang X, Zhang F, Gao Y, et al. A proteomic view of *Caenorhabditis elegans* caused by short-term hypoxic stress. Proteome Science. 2010;8: 49.

5. Honnen SJ, Buchter C, Schroder V, Hoffmann M, Kohara Y, et al. *C. elegans* VANG-1 Modulates Life Span via Insulin/IGF-1-Like Signaling. PLoS ONE. 2012;7: e32183.

6. Arda HE, Taubert S, MacNeil LT, Conine CC, Tsuda B, Van Gilst M, et al. Functional modularity of nuclear hormone receptors in a *Caenorhabditis elegans* metabolic gene regulatory network. Mol Syst Biol. 2010;6: 367.

7. Chen Y, Baugh LR. Ins-4 and daf-28 function redundantly to regulate *C. elegans* L1 arrest. Developmental Biology. 2014;394: 314–326.

8. Tullet JM, Hertweck M, An JH, Baker J, Hwang JY, Liu S, et al. Direct Inhibition of the Longevity-Promoting Factor SKN-1by Insulin-like Signaling in *C. elegans.* Cell. 2008;132: 1025-38.
9. Beale EG. 5'-AMP-Activated Protein Kinase Signaling in *Caenorhabditis elegans.* Exp Biol Med (Maywood). 2008;233: 12-20.

10. Liu M, Liu P, Zhang L, Cai Q, Gao G, Zhang W. mir-35 is involved in intestine cell G1/S transition and germ cell proliferation in *C. elegans.* Cell Research. 2011;21: 1605-1618.
11. Mapes J, Chen JT, Yu JS, Xue D. Somatic sex determination in *Caenorhabditis elegans* is modulated by SUP-26 repression of tra-2 translation. *Proc Natl Acad Sci U S A.* 2010;*107: 18022-7.*
12. Piano F, Schetter AJ, Morton DG, Gunsalus KC, Reinke V, Kim SK, et al. Gene clustering based on RNAi phenotypes of ovary-enriched genes in *C. elegans.* Curr Biol. 2002;12: 1959-64.

13. Cheng KC, Klancer R, Singson A, Seydoux G. Regulation of MBK-2/DYRK by CDK-1 and the Pseudophosphatases EGG-4 and EGG-5 during the Oocyte-to-Embryo Transition. Cell. 2009;139: 560-72.

14. Heschl MF, Baillie DL. The hsp70 multigene family of *Caenorhabditis elegans.* Comp Biochem Physiol B. 1990;96: 633-7.

15. Karp X, Ambros V. The Developmental Timing Regulator hbl-1 Modulates the Dauer Formation Decision in *Caenorhabditis elegans* Genetics. 2011;187: 345-53.

16. Maduro MF, Rothman JH. Making Worm Guts: The Gene Regulatory Network of the *Caenorhabditis elegans* Endoderm. Dev Biol. 2002;246: 68-85.
17. Segbert C, Johnson K, Theres C, van Fürden D, Bossinger O. Molecular and functional analysis of apical junction formation in the gut epithelium of *Caenorhabditis elegans*. Dev Biol. 2004;266: 17-26.

18. Schouest KR, Kurasawa Y, Furuta T, Hisamoto N, Matsumoto K, et al. The Germinal Center Kinase GCK-1 Is a Negative Regulator of MAP Kinase Activation and Apoptosis in the *C. elegans* Germline. PLoS ONE. 2008;4: e7450.
19. Kuznicki KA, Smith PA, Leung-Chiu WM, Estevez AO, Scott HC, Bennett KL. Combinatorial RNA interference indicates GLH-4 can compensate for GLH-1; these two P granule components are critical for fertility in *C. elegans.* Developmen. 2000;127: 2907-16.
20. Petcherski AG, Kimble J. LAG-3 is a putative transcriptional activator in the *C. elegans* Notch pathway. Nature. 2000;405: 364-8.
21. Harrison MM, Ceol CJ, Lu X, Horvitz HR. Some *C. elegans* class B synthetic multivulva proteins encode a conserved LIN-35 Rb-containing complex distinct from a NuRD-like complex. Proc Natl Acad Sci U S A. 2006;103: 16782-7.
