# Supplementary material for: High-Throughput Profiling of Caenorhabditis elegans Starvation-Responsive microRNAs
Source: PLoS One. 2015 Nov 10;10(11):e0142262. doi: 10.1371/journal.pone.0142262 (PMC4640506; doi:10.1371/journal.pone.0142262)
Supplement: S1 Table — Primers used for Reverse Transcription and qPCR. The Universal ProbeLibrary Probe #21 binding sites are highlighted in red. (PDF) [file pone.0142262.s002.pdf]

S1 Table. List of primers. Primers used for Reverse Transcription and qPCR

Name	Sequence
**RTCEL35**	GTT GGC TCT GGT GCA GGG TCC GAG GTA TTC GCA CCA GAG CCA ACA CTG CT
**FCEL35**	GCG GCG GTC ACC GGG TGG AAA CT
**RTCEL36**	GTT GGC TCT GGT GCA GGG TCC GAG GTA TTC GCA CCA GAG CCA ACC ATG CG
**FCEL36**	GCG GCG GTC ACC GGG TGA AAA TT
**RTCEL39**	GTT GGC TCT GGT GCA GGG TCC GAG GTA TTC GCA CCA GAG CCA ACC AAG CT
**FCEL39**	GCG GCG GTC ACC GGG TGT AAA TC
**RTCEL240**	GTT GGC TCT GGT GCA GGG TCC GAG GTA TTC GCA CCA GAG CCA ACG CAT TC
**FCEL240**	GCG GCG GCG AGG ATT TTG AGA CTA
**RTCEL246**	GTT GGC TCT GGT GCA GGG TCC GAG GTA TTC GCA CCA GAG CCA ACG CTC CT
**FCEL246**	GCG GCG GTT ACA TGT TTC GGG T
**RTCEL58**	GTT GGC TCT GGT GCA GGG TCC GAG GTA TTC GCA CCA GAG CCA ACA TTG CC
**FCEL58**	GCG GCG GTG AGA TCG TTC AGT AC
**UPR**	GTG CAG GGT CCG AGG T
**LIN23-forward**	TGT TCC GCC TGC AGT TTG A
**LIN23-reverse**	GTC CAA AAA GTC CCA AAT GAG AA
**GLD-forward**	GGA AAG TGC TCA CCG TGG AA
**GLD-reverse**	TGT TCG AGT GCT GCT TGC A
**ACT-forward**	CCG TGA AAA GAT GAC CCA AAT C
**ACT-reverse**	GGT ACG TCC GGA AGC GTA GA

The Universal ProbeLibrary Probe #21 binding sites are highlighted in red.
