# Supplementary material for: High-Throughput Profiling of Caenorhabditis elegans Starvation-Responsive microRNAs
Source: PLoS One. 2015 Nov 10;10(11):e0142262. doi: 10.1371/journal.pone.0142262 (PMC4640506; doi:10.1371/journal.pone.0142262)
Supplement: S2 Table — Targets with known metabolic functions predicted for differentially expressed miRNAs. (PDF) [file pone.0142262.s003.pdf]

S2 Table. Predicted miRNA targets with metabolic functions. Targets with known metabolic functions predicted for differentially expressed miRNAs.

microRNA	Predicted mRNA target	Function	Reference
miR-35-3p	*egl-3*	EGL-3, a prohormone convertase that processes agonistic Insulin/ IGF ligands INS-4 and INS-6, in neurons.	[1]
let-7-3p	*gsy-1*	Orthologous to the human gene GLYCOGEN SYNTHASE 1, is the rate- limiting enzyme in the synthesis of glycogen, which is likely to play a role in desiccation survival.	[2]
miR-4813-5p	*B0301.1*	Encodes a nematode-specific transmembrane protein; loss of B0310.1 activity via RNAi results in reduced fat content in wild-type and *tub-1* mutant animals, suggesting that B0301.1 plays a role in lipid metabolism.	[3]
miR-34-3p	*atp-5*	ATP synthase subunit 5, mitochondrial.	[4]
miR-41-5p	*vang-1*	Involved in lifespan, *vang-1* mutants show defects like reduced brood size, decreased ovulation rate and prolonged reproductive span, which are also related to gerontogenes. Lifespan extension in *vang-1* mutants depends on the Insulin\/IGF-1-like receptor DAF-2 and DAF-16/FoxO transcription factor.	[5]
miR-85-5p	*nhr-28*	NHRs are metabolic sensors, in particular, *nhr-28* is involved in lipid storage.	[6]
miR-39-5p	*ins-9*	Encodes a putative insulin-like peptide, insulin-like genes are expressed in response to nutrient availability and are involved in post-embryonic development.	[7]
miR-240-5p	*skn-1*	SKN-1 defends against oxidative stress by mobilizing the conserved phase-2 detoxification response. The Insulin/IGF-1-like signaling (IIS) kinases AKT-1, AKT-2, and SGK-1 phosphorylate SKN-1, and reduced IIS leads to constitutive SKN-1 nuclear accumulation in the intestine and SKN-1 target gene activation. The transcription network regulated by SKN-1 promotes longevity and is an important direct target of IIS.	[8]
miR-246-3p	*aakg-1*	Encodes one of five *C. elegans* AMP kinase (AMPK) gamma regulatory subunits.	[9]
