# Supplementary material for: High-Throughput Profiling of Caenorhabditis elegans Starvation-Responsive microRNAs
Source: PLoS One. 2015 Nov 10;10(11):e0142262. doi: 10.1371/journal.pone.0142262 (PMC4640506; doi:10.1371/journal.pone.0142262)
Supplement: S3 Table — Targets with known developmental functions predicted for differentially expressed miRNAs. (PDF) [file pone.0142262.s004.pdf]

**S3 Table. Predicted miRNA targets with developmental functions.** Targets with known developmental functions predicted for differentially expressed miRNAs.

| microRNA | Predicted mRNA target | Function | Reference |
|---|---|---|---|
| miR-35-3p | ***lin-23*** | Regulates cell proliferation. | [10] |
| | ***gld-1*** | Plays a key role in determining meiosis entry and indirectly regulates mitotic proliferation of the germline. | [10] |
| | ***sup-26*** | SUP-26 associates with poly (A)-binding protein 1 (PAB-1) in vivo and may repress *tra-2* expression by inhibiting the translation-stimulating activity of PAB-1. Together, mRNA-binding factors repress translation and modulate sexual development in different tissues of *C. elegans*. | [11] |
| let-7-3p | ***nasp-2*** | Encodes an ortholog of human nuclear autoantigenic sperm protein (histone-binding) that is involved in embryo development and locomotion. | [12] |
| | ***mbk-2*** | Promote the oocyte-to-embryo transition. | [13] |
| miR-4813-5p | ***hsp-3*** | HSP-3 likely functions as a molecular chaperone, and is expressed constitutively (expression is not heat inducible) throughout development with greatest abundance during the L1 larval stage. | [14] |
| miR-34-3p | ***hbl-1*** | Key transcriptional regulator of L2 *vs.* L3 cell fate, can also modulate the dauer formation decision in a complex manner. | [15] |

| | | | |
|---|---|---|---|
| **miR-41-5p** | ***vang-1*** | Involved in lifespan, *vang-1* mutants show defects like reduced brood size, decreased ovulation rate and prolonged reproductive span, which are also related to gerontogenes. Lifespan extension in *vang-1* mutants depends on the Insulin/IGF-1-like receptor DAF-2 and DAF-16/FoxO transcription factor. | [5] |
| **miR-85-5p** | ***dlg-1*** | Its product is physically located at apical adherens junctions in all epithelia and is required for organization of the embryonic gut epithelium into a coherent tube. | [17] |
| **miR-39-5p** | ***gck-4*** | The germinal center kinases (GCK) constitute a large, highly conserved family of proteins that have been implicated in a wide variety of cellular processes including cell growth and proliferation, polarity, migration, and stress responses. | [18] |
| **miR-240-5p** | ***glh-2*** | Plays a role in germ-line development and fertility, specifically in regulating the normal extent of germ-line proliferation, oogenesis, and the production of functional sperm. | [19] |
| **miR-246-3p** | ***lin-37*** | Involved in nematode larval development, embryo development, reproduction and negative regulation of vulval development; LIN-37 is localized to the nucleus. | [21] |
